# Functional Characterization of Entamoeba histolytica Argonaute Proteins Reveals a Repetitive DR-Rich Motif Region That Controls Nuclear Localization

**DOI:** 10.1128/mSphere.00580-19

**Published:** 2019-10-16

**Authors:** Hanbang Zhang, Vy Tran, Dipak Manna, Gretchen Ehrenkaufer, Upinder Singh

**Affiliations:** a Division of Infectious Diseases, Department of Internal Medicine, Stanford University School of Medicine, Stanford, California, USA; b Department of Microbiology and Immunology, Stanford University School of Medicine, Stanford, California, USA; University at Buffalo

**Keywords:** Argonaute proteins, *Entamoeba*, RNA interference, gene expression, transcriptional regulation

## Abstract

The RNA interference (RNAi) pathway regulates gene expression in many eukaryotic organisms. Argonaute (Ago) proteins, together with bound small RNAs (sRNAs), are key effectors that mediate gene silencing function. However, there is limited knowledge of Ago proteins and their functions in nonmodel systems. In the protozoan parasite Entamoeba histolytica, RNAi is a robust means for stable gene silencing mediated via large populations of antisense sRNAs. Here, we report functional characterization of three Ago proteins in E. histolytica (*Eh*Ago2-1, *Eh*Ago2-2, and *Eh*Ago2-3). Our data show that each *Eh*Ago protein has a distinct subcellular localization and binds 27-nucleotide (nt) sRNAs and that the localization of *Eh*Ago proteins is altered in response to stress conditions. Via mutagenesis analyses, we demonstrated that the Ago PAZ (Piwi/Argonaute/Zwille) domain in all three *Eh*Agos is essential for sRNA binding. With mutation of the PAZ domain in *Eh*Ago2-2, there was no effect on the nuclear localization of the protein but a strong phenotype and a growth defect. We further show that *Eh*Ago2-2 contains an unusual repetitive DR-rich (aspartic acid, arginine-rich) motif region which functions as a nuclear localization signal (NLS) and is both necessary and sufficient to mediate nuclear localization. Overall, our data delineate the localization and sRNA binding features of the three E. histolytica Ago proteins and demonstrate that the PAZ domain is necessary for sRNA binding. The repetitive DR-rich motif region in *Eh*Ago2-2 has not previously been defined in other systems, which adds to the novel observations that can be made when studies of the RNAi pathway are extended to nonmodel systems.

**IMPORTANCE** The protozoan parasite Entamoeba histolytica, which causes amebiasis and affects over 50 million people worldwide, contains an important RNAi pathway for gene silencing. Gene silencing via the RNAi pathway is mediated by the Argonaute (Ago) proteins. However, we lack knowledge on Ago function(s) in this nonmodel system. In this paper, we discovered that three E. histolytica Ago proteins (*Eh*Ago2-1, *Eh*Ago2-2, and *Eh*Ago2-3) all bind 27-nt small RNAs and have distinct subcellular localizations, which change in response to stress conditions. The *Eh*Agos bind small RNA populations via their PAZ domains. An unusual repetitive DR-rich motif region is identified in *Eh*Ago2-2 that functions as a nuclear localization signal. Our results show for the first time an active nuclear transport process of the *Eh*Ago2-2 RNA-induced silencing complex (RISC) in this parasite. These data add to the novel observations that can be made when studies of the RNAi pathway are extended to nonmodel systems.

## Abstract (precis)

The protozoan parasite Entamoeba histolytica, which causes amebiasis and affects over 50 million people worldwide, contains an important RNAi pathway for gene silencing. Gene silencing via the RNAi pathway is mediated by the Argonaute (Ago) proteins. However, we lack knowledge on Ago function(s) in this nonmodel system. In this paper, we discovered that three E. histolytica Ago proteins (*Eh*Ago2-1, *Eh*Ago2-2, and *Eh*Ago2-3) all bind 27-nt small RNAs and have distinct subcellular localizations, which change in response to stress conditions. The *Eh*Agos bind small RNA populations via their PAZ domains. An unusual repetitive DR-rich motif region is identified in *Eh*Ago2-2 that functions as a nuclear localization signal. Our results show for the first time an active nuclear transport process of the *Eh*Ago2-2 RNA-induced silencing complex (RISC) in this parasite. These data add to the novel observations that can be made when studies of the RNAi pathway are extended to nonmodel systems.

## INTRODUCTION

Argonaute (Ago) proteins and their bound small RNAs (sRNAs) are central to the RNA interference (RNAi) pathway, which mediates both posttranscriptional and transcriptional gene silencing (PTGS/TGS) in many eukaryotes (1–3). The RNAi pathway involves an sRNA-guided Ago complex, i.e., RNA-induced silencing complex (RISC), to target mRNA for degradation (small interfering RNA [siRNA]-based function), translational repression (microRNA [miRNA]-based function), or chromatin modification (epigenetic silencing function) (4). The complexity and plasticity of RNAi pathways have been demonstrated in higher eukaryotes, which often contain multiple Ago proteins with different sRNA classes (siRNA, miRNA, Piwi-interacting RNA [piRNA], secondary siRNA, etc.) (5). For example, there are eight Ago proteins in Homo sapiens, 10 Ago proteins in Arabidopsis thaliana, and 27 Ago proteins in Caenorhabditis elegans (6). In contrast, single-celled eukaryotes often show a simplified RNAi pathway with fewer Ago proteins. As an example, the yeast Schizosaccharomyces pombe has a single Ago protein (7), whereas no RNAi pathway is found in the related yeast Saccharomyces cerevisiae (7). Among the unicellular protists, the RNAi pathway is often less well studied; Ago proteins exist in Trypanosoma brucei (two Ago proteins), Toxoplasma gondii (three Ago proteins), Giardia lamblia (one Ago protein), and Trichomonas vaginalis (two Ago proteins) but are absent in *Plasmodium* and Trypanosoma cruzi (6, 8). Small RNA sequencing and experimental data for these organisms revealed functional RNAi pathways for retrotransposon control, gene regulation, and antigenic variation (6, 8).

The protozoan parasite Entamoeba histolytica causes amebiasis and is a major health concern in developing countries (9, 10). The parasite has two life stages: a dormant cyst form and an infective and invasive trophozoite form. The E. histolytica genome encodes several key RNAi machinery components, including three Ago proteins (*Eh*Ago2-1 [EHI_186850], *Eh*Ago2-2 [EHI_125650], and *Eh*Ago2-3 [EHI_177170]), three RNA-dependent RNA polymerases (RdRPs) (EHI_139420, EHI_179800, and EHI_086260), and one protein (EHI_068740) with a single RNase III domain that has double-stranded RNA (dsRNA) cleavage activity but without a dsRNA binding domain (dsRBD) or other domains typically found in canonical Dicer enzymes (11, 12). We have previously reported that E. histolytica *Eh*Ago2-2 has nuclear localization and binds to an atypical 27-nucleotide (nt) sRNA population that has 5′-polyphosphate (polyP) structure (13). We further linked these 27-nt sRNAs with endogenous gene silencing and developed an RNAi-based trigger method to silence genes of interest in this parasite (14). We demonstrated that RNAi-induced TGS in E. histolytica is established via histone modification at H3K27 and with the association of *Eh*Ago2-2 protein (15). Furthermore, 27-nt sRNAs in *Entamoeba* are involved in regulation of strain-specific virulence genes but do not appear to regulate stage conversion between the trophozoite and cyst stages or the amebic stress response to heat shock or oxidative stress (16, 17). Our attempts using an RNAi-based trigger method to silence the three *Eh*Ago genes failed to downregulate any of these transcripts, despite the fact that antisense sRNAs could be detected in parasites (18), suggesting that these genes may be essential and/or resistant to RNAi-mediated gene silencing. As to EHI_068740, our attempts using an *in vitro* dsRNA cleavage assay failed to show cleavage activity of this protein under standard experimental conditions (11). However, it partially contributes gene silencing in a heterologous system (S. cerevisiae) along with *Sca*Ago1 (11).

In this study, we have further characterized the three Ago proteins in E. histolytica. We demonstrated that the three *Eh*Ago proteins have distinct subcellular localizations and all bind 27-nt sRNAs. We showed that expression and localization of *Eh*Ago proteins change in response to two stress conditions, indicating a possible role for gene regulation under these conditions. We dissected the PAZ domain function in *Eh*Ago by mutational analysis and demonstrated that *Eh*Ago binding to sRNA was abolished/severely affected upon PAZ mutations; lack of sRNA binding in turn altered cellular localization of *Eh*Ago2-1 and *Eh*Ago2-3 but not *Eh*Ago2-2. Furthermore, we identified an unusual repetitive DR-rich motif region in *Eh*Ago2-2, not previously seen in other systems, which is necessary and sufficient to mediate nuclear localization. Our data provide the first functional analyses of the three E. histolytica Ago proteins, including the novel nuclear localization signal (NLS) function of the repetitive DR-rich motif region in *Eh*Ago2-2, which adds to the known diversity of the structural and functional roles of Ago family proteins.

## RESULTS

### *Eh*Ago proteins have conserved structural PAZ and PIWI domains.

**FIG 1:**
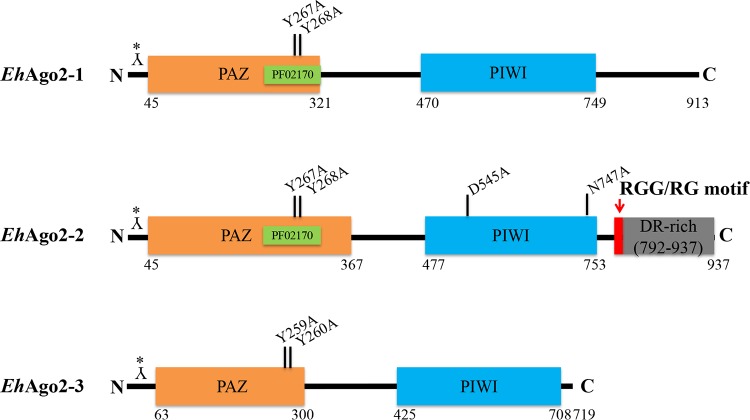
The structural domains (PAZ and PIWI) of three *Eh*Ago proteins in E. histolytica. The length of proteins is drawn to scale, with the length of PAZ and PIWI based on the annotation of AmoebaDB. We generated *Eh*Ago polyclonal antibodies in rabbits using peptide from the N-terminal end of sequence as indicated by Y* (see also Materials and Methods). The PAZ Superfam domain (SSF101690) is shown in orange, and the PAZ Pfam (PF02170) domain of *Eh*Ago2-1 and *Eh*Ago2-2 is shown in green. The PIWI Pfam (PF02171) domain is in blue. Specific PAZ/PIWI mutations generated in this study are shown. Both *Eh*Ago2-1 and *Eh*Ago2-2 have ∼200 aa after the PIWI domain, with an RGG/RG motif (red) and repetitive DR-rich region (gray) identified in *Eh*Ago2-2.

RNAi gene regulation pathways and Ago proteins have been found across most eukaryotes (19). Ago proteins are generally conserved for four structural domains (the N terminus, PAZ, middle, and PIWI domains) (20). The E. histolytica genome contains genes encoding three Ago family proteins, *Eh*Ago2-1 (EHI_186850), *Eh*Ago2-2 (EHI_125650), and *Eh*Ago2-3 (EHI_177170) (21), with all three Ago proteins showing the conserved PAZ and PIWI domains (Fig. 1). Although the PAZ Superfam domain is annotated for all three *Eh*Agos, the PAZ Pfam PF02170 domain is not identified for *Eh*Ago2-3, probably due to its sequence divergence. Our phylogenetic analysis using the current genomic data set of AmoebaDB for species including Entamoeba moshkovskii, Entamoeba dispar, Entamoeba nuttalli, and Entamoeba invadens indicates that all three *Eh*Agos are conserved among these amebic species, and each *Eh*Ago forms its own cluster. *E. moshkovskii* and *E. invadens* are more divergent than the other three species within each cluster (see Fig. S1 in the supplemental material). Evolutionary loss of RNAi can occur in some eukaryote taxa, such as yeast Saccharomyces castellii (Ago and RNAi positive) versus S. cerevisiae (Ago and RNAi negative) (7) and T. brucei (Ago and RNAi positive) versus T. cruzi (Ago and RNAi negative) (22). Our analysis of current genomes of ameba species indicated that the RNAi pathway(s) is well conserved in these amebic species. Thus, elucidation of biological functions of Ago proteins is important to understanding the biology and pathogenesis of this unicellular parasite.

10.1128/mSphere.00580-19.1 FIG S1 Phylogenetic analysis of Agos among five amebic species. The five species are E. histolytica HM-1:IMSS, *E. nuttallii* P19, *E. invadens* IP1, *E. moshkovskii* Laredo, and *E. dispar* SAW760. Full-length sequences of Ago were used to build a phylogenetic tree using an online phylogeny tool (http://www.phylogeny.fr) using default settings. As shown, three *Eh*Agos form clusters among the five species. As noted, we combined EIN_309880 and EIN_309720 as one Ago, due to our observation that the current genome has a gap between these two truncated genes (data not shown). Download FIG S1, TIF file, 0.1 MB. Copyright © 2019 Zhang et al. 2019 Zhang et al. This content is distributed under the terms of the Creative Commons Attribution 4.0 International license.

### The three *Eh*Ago proteins have distinct subcellular localizations.

**FIG 2:**
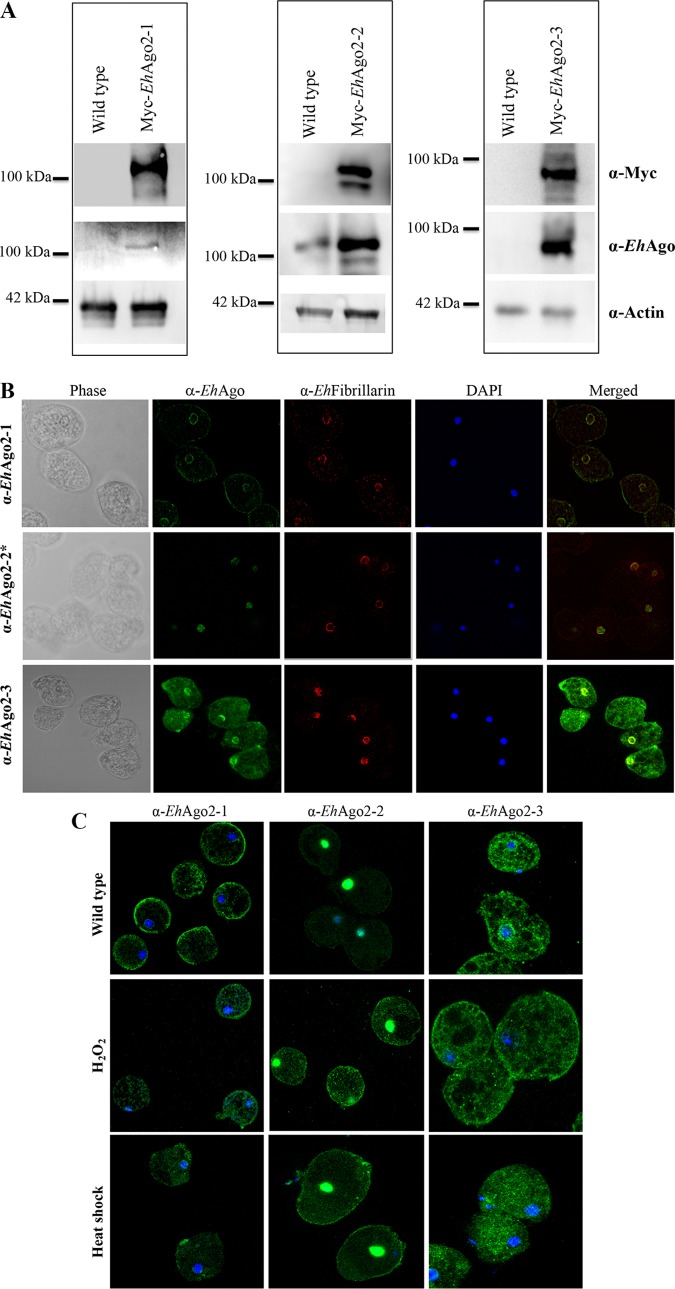
IFAs for the three *Eh*Agos show distinct subcellular localization in the parasite, and stress conditions affect their expression/localization. (A) Custom-made antibodies to the three *Eh*Agos detect specific bands by Western blotting. Western blot analyses were performed on total cell lysates from wild-type and Myc-tagged overexpressing cell lines. The expected band size of *Eh*Ago2-2 was detected in both lysate sample lanes; however, *Eh*Ago2-1 and *Eh*Ago2-3 can be detected only in lysates from overexpressing cell lines and not in wild-type cell lysates, likely due to their low endogenous expression, based on three published data sets using Affymetrix microarray (24, 25) or RNA-seq (26). Anti-actin was used as a loading control. (B) Distinct subcellular localizations are shown for each *Eh*Ago in the parasite by IFAs. E. histolytica trophozoites were fixed and immunostained using custom peptide antibodies for *Eh*Ago2-1, *Eh*Ago2-2, and *Eh*Ago2-3. Localization for *Eh*Ago2-2 has previously been published (23) and was used as a control. A ring structure at the perinuclear location was seen for both *Eh*Ago2-1 and *Eh*Ago2-3, contrasting with the nuclear staining for *Eh*Ago2-2 IFA. Additional signal was seen for *Eh*Ago2-1 (cell surface membrane) and for *Eh*Ago2-3 (cytosol). Anti-*Eh*Fibrillarin is used as a marker of the nucleolus, which is located at the nuclear periphery in E. histolytica. DAPI, 4′,6-diamidino-2-phenylindole. (C) Localization of *Eh*Ago proteins changes in response to stress conditions. IFAs of merged image of DAPI and each anti-*Eh*Ago are shown for untreated parasites and parasites treated with two stress conditions (42°C for 1 h or 1 mM H_2_O_2_ for 1 h). Compared with the wild-type condition, the *Eh*Ago2-1 localization of the perinuclear ring was almost lost under H_2_O_2_ treatment but was largely unchanged with heat shock. The *Eh*Ago2-2 signal completely overlapped with the DAPI signal in untreated parasites but was greatly increased throughout the cytoplasm under both stress conditions, along with noticeable punctate dots, but its nuclear localization signal remained for both stress conditions. The *Eh*Ago2-3 perinuclear localization is no longer observed under both stress conditions, and the major signal of staining is from cytoplasm. All stress condition assays were performed with multiple biological replicates and gave reproducible results; representative images are shown.

Previously, we have reported that *Eh*Ago2-2 is localized in the nucleus (23). To characterize *Eh*Ago2-1 and *Eh*Ago2-3, we generated custom-made polyclonal antibodies using selected N-terminal peptide sequences (Fig. 1). The antibodies were first tested by Western blot analysis using total cell lysates from wild-type and Myc-tagged overexpressing cell lines. The expected band size of each Ago was detected (Fig. 2A). However, due to low endogenous expression of both *Eh*Ago2-1 and *Eh*Ago2-3 (based on three published data sets using Affymetrix microarray [24, 25] or transcriptome sequencing [RNA-seq] [26], both *Eh*Ago2-1 and *Eh*Ago2-3 mRNA expression levels are detectable but are significantly lower than that of *Eh*Ago2-2), they could not be detected in wild-type cell lysates but only in lysates from overexpressing cell lines (Fig. 2A). We then performed immunostaining of trophozoites using these antibodies along with their preimmune sera. Immunofluorescence assay (IFA) with preimmune sera showed no specific signal at the background level (data not shown), but IFA with *Eh*Ago2-1 antibody revealed two predominant subcellular localizations, perinuclear ring and cell surface membrane; staining with *Eh*Ago2-3 antibody shows intense signal in both perinuclear and cytosolic localizations (Fig. 2B). Anti-*Eh*Fibrillarin is used to label the nucleolus, which was shown to be located at the nuclear periphery in E. histolytica (27). Localization of *Eh*Ago2-2 has been previously shown to be in the nucleus (23) and is included for comparison.

In model systems, Ago proteins are found to be localized to many cellular loci depending on their biological functions, including cytoplasm (P-bodies and stress granules) for their PTGS-related functions and perinuclear regions or nucleus for TGS-related functions (20). In Neurospora crassa, RNAi components including RdRP, Dicer, and Ago are localized in the perinuclear region, where they function in meiotic silencing by unpaired DNA events (28, 29). In C. elegans, Ago proteins CSR-1, WAGO-1, and PRG-1 are localized to germ line perinuclear nuage, where epigenetic inheritance factors interact with Ago and RdRP (30, 31). Our results show that the three *Eh*Ago proteins cover all major loci (cytoplasm, nucleus, and perinucleus), and each *Eh*Ago has a unique localization, indicating that *Entamoeba* could possess diverse RNAi-related roles with each *Eh*Ago protein having a unique function in the different aspects of RNAi.

### Expression and localization of *Eh*Ago proteins change in response to stress conditions.

In human cells, hAgo2 is localized mainly in the cytoplasm and concentrated in P-bodies and is rapidly distributed to stress granules upon stresses (32), indicating a dynamic intracellular localization change of Ago with stresses. In *E. histolytica*, both cytoplasmic granules under stress condition and P-body-like structures have been reported (33, 34), and our previous transcriptional profiling showed high-level gene expression changes in E. histolytica after heat shock and oxidative stress (35, 36). We therefore used fluorescence microscopy to study accumulation/loss of expression of the three *Eh*Agos under two conditions (42°C for 1 h or 1 mM H_2_O_2_ for 1 h).

The overall changes to the localization of endogenous *Eh*Ago proteins under these conditions are shown in Fig. 2. For *Eh*Ago2-1, the perinuclear ring in untreated cells was almost lost under H_2_O_2_ treatment but was unchanged with heat shock. With *Eh*Ago2-2, the nuclear localization remained for both untreated and stress-treated cells; however, signal was greatly increased throughout the cytoplasm under stress conditions compared to almost no cytoplasmic signal in untreated cells (Fig. 2C). There are also some distinct punctate dots with induction of oxidative stress, similar to the stress granules reported earlier for *E. histolytica* (33). However, due to the lack of a marker for these granules, we cannot definitively say if the *Eh*Ago staining overlaps. To provide confirmation for the increased *Eh*Ago2-2 protein levels in the cytoplasm upon stress, we performed cell fractionation for nuclear and cytoplasmic proteins based on an earlier method (37). Western blot analysis using anti-*Eh*Ago2-2 showed increased cytoplasmic signal compared with untreated cells (see Fig. S2 in the supplemental material). For *Eh*Ago2-3, the perinuclear localization in untreated cells was completely lost under both stress conditions, and the majority of staining was in the cytoplasm. RNAi machinery and its gene regulation mechanism have been indicated for many biological events (38), and Ago can interact with diverse proteins and protein complexes, including RNA processing, maturation, and transport and the regulation of RNA stability and translation (2). Our data indicated that RNAi machinery actively responds to stresses in amebae. However, earlier sequencing of 27-nt sRNA libraries for parasites under these two conditions showed rather similar profiles of sRNA species (17). This may indicate that *Eh*Ago localization changes help mediate changes in gene expression as a response to stress.

10.1128/mSphere.00580-19.2 FIG S2 Expression of *Eh*Ago2-2 proteins changes in response to H_2_O_2_ stress condition. Cell fractionation samples for cytoplasmic and nuclear lysates were made for both untreated and stress-treated conditions. Western blot assays with anti-*Eh*Ago2-2 show a noticeable band for *Eh*Ago2-2 in the cytoplasmic sample under the H_2_O_2_ stress condition. Anti-actin was used as a loading control, and anti-histone H3 was used as a marker for the nuclear fraction. Download FIG S2, PDF file, 1.3 MB. Copyright © 2019 Zhang et al. 2019 Zhang et al. This content is distributed under the terms of the Creative Commons Attribution 4.0 International license.

### The three *Eh*Ago proteins show specific sRNA binding that is abolished/severely degraded by YY-to-AA mutation in the PAZ domain.

Phylogenic analyses for eukaryotic Ago proteins have divided all Ago proteins into three clades: the Ago-like, the Piwi-like, and the WAGO subfamilies (6, 19, 39). Amebozoan Ago proteins are placed into a PIWI-like clade (6, 19). In order to identify conserved residues, we aligned three *Eh*Ago PAZ and PIWI domains with human HIWI and *Drosophila* PIWI domain sequences using the Clustal Omega tool (Fig. S3). It is well documented that the PAZ domain binds the 3′ end of sRNAs with some highly conserved residues, the so-called R/K-F-Y signature sites (20). The alignment of three *Eh*Ago PAZs showed that these signature residues are conserved for *Eh*Ago2-1 and *Eh*Ago2-2 as highlighted in the red box in Fig. S3, but not for *Eh*Ago2-3, for which the residues are W-K-Y (of note, two FF residues are right next to K, which may indicate that *Eh*Ago2-3 could have W-F-Y residues). In addition, the mutation of the two tyrosine residues in PAZ was shown to abolish sRNA binding in C. elegans (40). We therefore selected these two residues for mutagenesis as indicated in Fig. 1.

10.1128/mSphere.00580-19.3 FIG S3 Three *Eh*Ago PAZ domains share conserved residues with PAZ in higher eukaryotes (*Hs*HIWI and *Dm*PIWI). The PAZ domain sequences of three *Eh*Ago proteins along with PAZ sequences from H. sapiens HIWI and D. melanogaster PIWI are aligned using Clustal alignment (www.ebi.ac.uk/Tools/msa/clustalo/). The R/K-F-Y signature sites, highly conserved residues for binding the 3′ end of sRNAs, are boxed in red. Two tyrosine residues are mutated to alanine as indicated by solid black triangles. The positions of PAZs are as follows: *Eh*Ago2-1, 211 to 312; *Eh*Ago2-2, 209 to 307; *Eh*Ago2-3, 201 to 300; *Hs*HIWI, 277 to 391; *Dm*PIWI, 262 to 372. GenBank IDs are as follows: *Eh*Ago2-1, 3410768; *Eh*Ago2-2, 3410821; *Eh*Ago2-3, 3407129; *Hs*HIWI, 33563234; *Dm*PIWI, 17136736. Download FIG S3, PDF file, 0.08 MB. Copyright © 2019 Zhang et al. 2019 Zhang et al. This content is distributed under the terms of the Creative Commons Attribution 4.0 International license.

One characteristic of Ago proteins is the ability to bind sRNAs. We reported earlier that *Eh*Ago2-2 binds antisense 27-nt small RNAs and is linked to TGS in E. histolytica (13, 14). To check if sRNAs are also bound to *Eh*Ago2-1 and *Eh*Ago2-3, we overexpressed Myc-tagged protein for *Eh*Ago2-1 and *Eh*Ago2-3 using the same vector that was used for *Eh*Ago2-2 (13). Additionally, based on the sequence alignment of PAZ, we mutated the conserved YY to AA and generated stable overexpression transfectants for Myc-*Eh*Ago2-1 PAZ^mut^ and Myc-*Eh*Ago2-3 PAZ^mut^ mutants. We were not able to generate Myc-*Eh*Ago2-2 PAZ^mut^-stably transfected parasites after multiple attempts. We speculated that the overexpression of Myc-*Eh*Ago2-2 PAZ^mut^ could be harmful to parasite growth and viability. Thus, to minimize the potential toxicity of Myc-*Eh*Ago2-2 PAZ^mut^, we used a protein destabilization domain approach (41) to establish dihydrofolate reductase (DHFR)-Myc-*Eh*Ago2-2 PAZ^mut^ in parasites. The induction of DHFR-Myc-*Eh*Ago2-2 PAZ^mut^ protein was detected upon addition of a stabilizing agent (Fig. S4).

10.1128/mSphere.00580-19.4 FIG S4 Protein destabilization domain (DD) approach establishes regulated expression of Myc-DHFR-*Eh*Ago2-2 PAZ^mut^. Anti-Myc Western blotting showed the expected-size band for mutant protein upon TMP induction. Whole-cell lysates (50 μg protein) from trophozoites expressing N-terminal Myc-DHFR-*Eh*Ago2-2 PAZ^mut^ with and without added TMP were loaded. Anti-actin Western blotting was used as a loading control on the same membrane. Download FIG S4, PDF file, 0.09 MB. Copyright © 2019 Zhang et al. 2019 Zhang et al. This content is distributed under the terms of the Creative Commons Attribution 4.0 International license.

**FIG 3:**
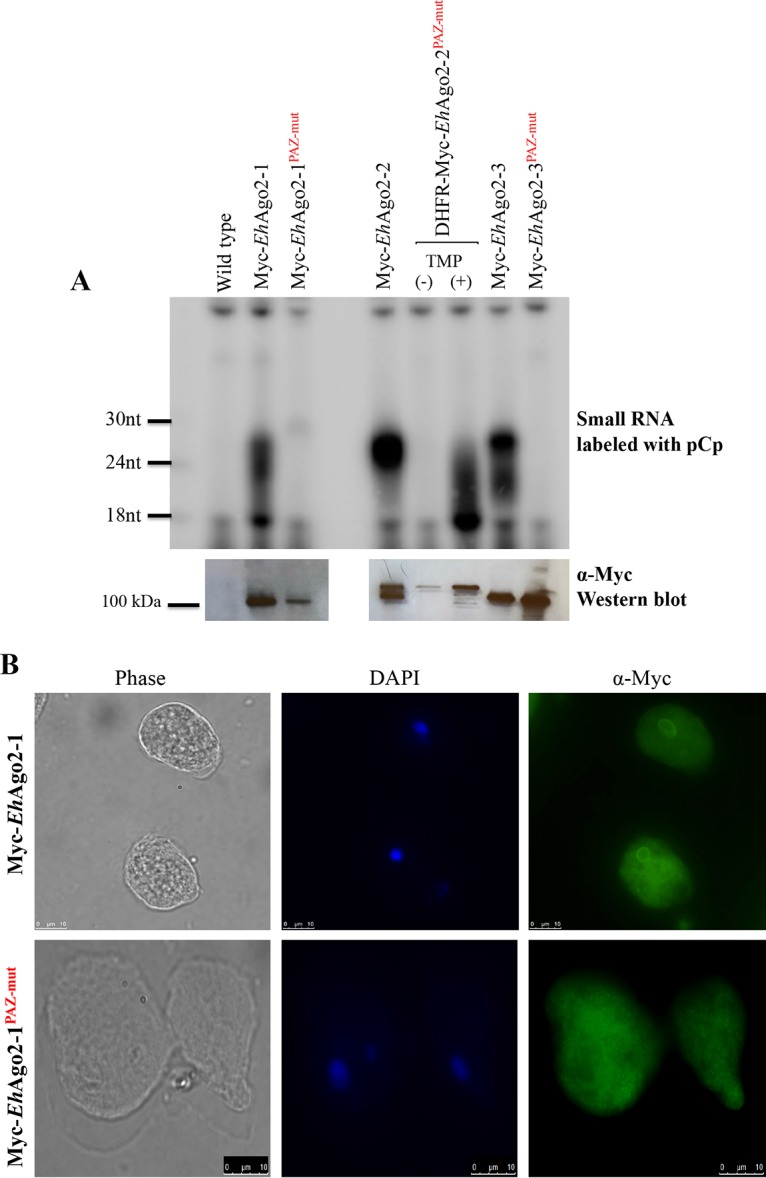

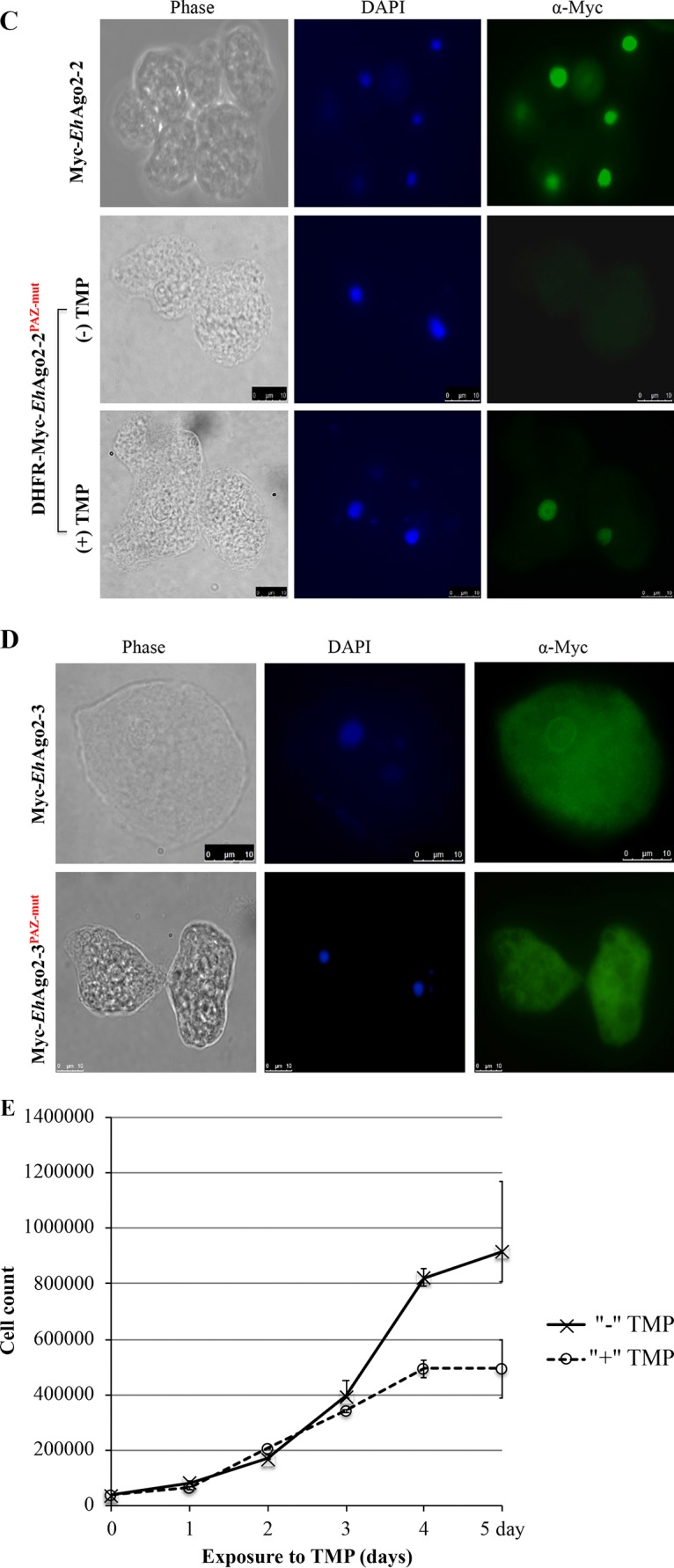
Characterization of three *Eh*Agos and their PAZ mutants for sRNA binding and protein localization. (A) Specific sRNA populations are bound to each Myc-*Eh*Ago, and PAZ domain mutations abolished or severely degraded sRNA binding. Overexpressing Myc-tagged cell lines of wild-type *Eh*Agos and PAZ mutants were used. For *Eh*Ago2-2 PAZ mutant, a protein destabilization domain approach was used to express mutant protein upon addition of TMP. Shown in the upper panel is the pCp-labeled IP sRNA, showing specific sRNA populations bound to each *Eh*Ago protein, which is absent in the control IP of the wild-type line or the uninduced Myc-DHFR-*Eh*Ago2-2 PAZ^mut^ line. While Myc-*Eh*Ago2-2 binds to a distinct 27-nt sRNA population, sRNA bands bound to Myc-*Eh*Ago2-1 and Myc-*Eh*Ago2-3 are rather smeary, composed of a noticeable 27-nt band along with its streak down to 20 nt. The PAZ mutations abolish the sRNA binding in Myc-*Eh*Ago2-1 PAZ^mut^ and Myc-*Eh*Ago2-3 PAZ^mut^. However, the 27-nt band in Myc-*Eh*Ago2-2 turned into a greatly degraded pattern in Myc-DHFR-*Eh*Ago2-2 PAZ^mut^. The lower panel is the anti-Myc Western blot analysis detecting specific Myc pulldown of Myc-*Eh*Ago as well as its counterpart for the PAZ mutant. Both IP RNA and IP protein samples were made from the same anti-Myc IP pulldown experiment by splitting beads into half-and-half samples as described in Materials and Methods. (B) Anti-Myc IFAs show that the perinuclear ring localization is lost for mutant Myc-*Eh*Ago2-1 PAZ^mut^ in comparison with wild-type Myc-*Eh*Ago2-1. (C) Anti-Myc IFAs with and without TMP demonstrate that the mutant Myc-DHFR-*Eh*Ago2-2 PAZ^mut^ has nuclear localization similar to wild-type Myc-*Eh*Ago2-2. (D) Anti-Myc IFAs show that the perinuclear ring localization is lost for mutant Myc-*Eh*Ago2-3 PAZ^mut^ in comparison with wild-type Myc-*Eh*Ago2-3. (E) Growth kinetics for the Myc-DHFR-*Eh*Ago2-2 PAZ^mut^ cell line. A significant reduction in the growth rate of the mutant cell line at days 4 and 5 was observed compared with the no-TMP control. Error bars are calculated as standard error from duplicate samples. We have extensively tested the effect of TMP on parasite growth in multiple *Entamoeba* strains and species and found no/minimal effect on growth rates (41).

We used the cell lines described above to determine sRNA binding. For all three *Eh*Ago Myc-tagged overexpressed cell lines and their PAZ mutants, we performed anti-Myc immunoprecipitation (IP) using whole-cell lysate. Both IP RNA and IP protein were collected; IP RNA was labeled by radioactive labeling after RNA separation on a denaturing gel; IP proteins were subjected to Western blot analysis. The data demonstrate that for Myc-*Eh*Ago2-1, the bound sRNAs form a smear at the 20- to 27-nt range, which disappears in the PAZ mutant (Fig. 3A). For Myc-*Eh*Ago2-2, a distinct 27-nt sRNA population is shown, as we previously observed (13); the PAZ mutation of *Eh*Ago2-2 shows no intact 27-nt sRNA but a severely degraded pattern of sRNA down to 18 nt. For Myc-*Eh*Ago2-3, there appear to be two sRNA populations at 27 nt and a smear of 20 to 24 nt, and upon generation of a PAZ mutant, there is no bound sRNA. Western blotting of IP proteins in the lower panels in Fig. 3A shows that wild-type and mutant lines pulled down similar levels of protein, indicating that the sRNA difference is not due to differences in IP efficiency or protein expression level. In conclusion, the Myc IP for three PAZ mutant *Eh*Ago lines failed to pull down specific sRNAs in *Eh*Ago2-1 and in *Eh*Ago2-3 mutants and severely degraded sRNA in the *Eh*Ago2-2 mutant, indicating that the PAZ domain provides both binding and protection for sRNA, possibly on its 3′ end. Without it, sRNA is subjected to exonucleases for either complete degradation (in the case of *Eh*Ago2-1 and *Eh*Ago2-3 PAZ mutants) or partial degradation (in the case of the *Eh*Ago2-2 PAZ mutant). These data confirm that PAZ RNA recognition is well conserved, and the binding mechanism of the 3′ end of sRNA of *Eh*Agos likely uses the same mechanism as all other Agos in model systems (42).

Further, we performed fluorescence microscopy assay for these cell lines and observed a significant change in the localization of mutant proteins in Myc-*Eh*Ago2-1 PAZ^mut^ and Myc-*Eh*Ago2-3 PAZ^mut^. The perinuclear localization of both Myc-*Eh*Ago2-1 and Myc-*Eh*Ago2-3 was absent in both PAZ^mut^ mutants (Fig. 3B and D). However, in DHFR-Myc-*Eh*Ago2-2 PAZ^mut^, the nuclear localization was unchanged compared to wild-type parasites (Fig. 3C). These data indicate that the PAZ domain is critical for sRNA binding for each of the three *Eh*Ago proteins and that the protein/sRNA binding appears to affect localization for *Eh*Ago2-1 and *Eh*Ago2-3 but not for *Eh*Ago2-2. As sRNA is a guide for RISC to find its target in different subcellular compartments, this may explain how PAZ mutants of *Eh*Ago2-1 and *Eh*Ago2-3 lost their specific protein localization. However, localization of the *Eh*Ago2-2 PAZ mutant is unchanged despite severely degraded sRNA, indicating that either severely degraded sRNA can still function as a guide for RISC or/and there is a specific nuclear importing process for this protein that functions through the NLS pathway.

### The *Eh*Ago2-2 PAZ mutant has a growth defect.

Given that we could not establish an *Eh*Ago2-2 PAZ mutant without using the destabilization domain approach, we wondered whether the *Eh*Ago2-2 PAZ mutation conferred a growth disadvantage on the parasites. The protein destabilization domain approach provides a way to study protein function for a short period of time upon adding a stabilizing agent. In order to identify the effect of the PAZ mutation on cell growth, we monitored the growth kinetics for the DHFR-Myc-*Eh*Ago2-2 PAZ^mut^ cell line upon adding the stabilizing agent trimethoprim (TMP) and inducing the PAZ mutant protein. We observed a significant reduction in the growth rate of the DHFR-Myc-*Eh*Ago2-2 PAZ^mut^ cell line at days 4 and 5 compared to dimethyl sulfoxide (DMSO) controls (Fig. 3E). Of note, the stabilization of *Eh*Ago2-2 PAZ mutant protein is in the context of endogenous wild-type *Eh*Ago2-2 expression. We have extensively tested TMP on parasite growth on different lines of culture and found no/minimal growth reduction (41); thus, the growth phenotype indicates that DHFR-Myc-*Eh*Ago2-2 PAZ^mut^ may be functioning in a dominant negative manner when overexpressed. Further characterization of the amebic RISC will shed light on the mechanistic action of *Eh*Ago2-2 function.

### *Eh*Ago2-2 has an unusual repetitive DR-rich motif that mediates nuclear localization.

**FIG 4:**
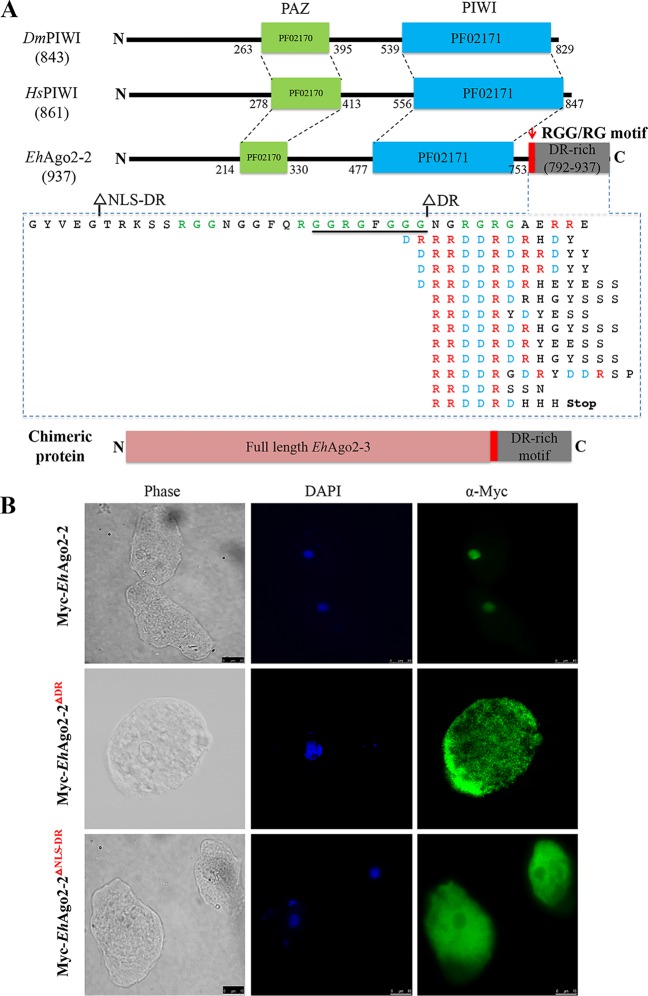

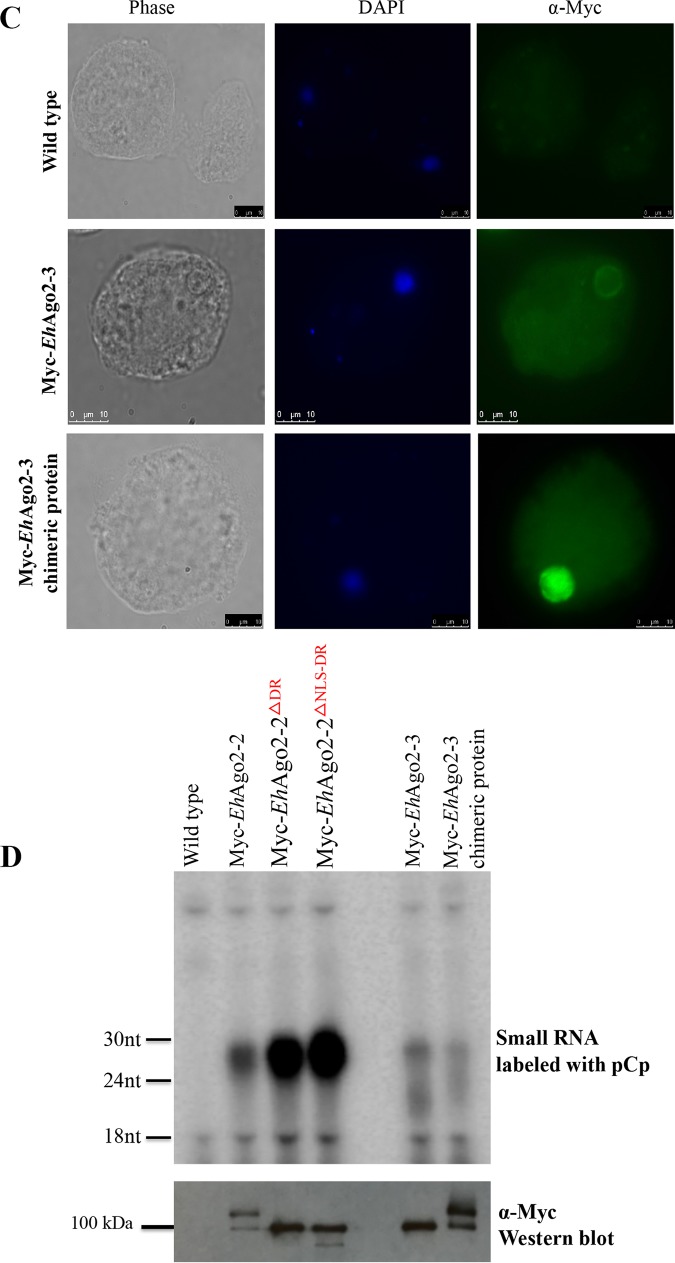
An unusual repetitive DR-rich motif region in *Eh*Ago2-2 mediates nuclear localization. (A) The C-terminal repetitive DR-rich motif region in *Eh*Ago2-2. Structural domains (PAZ and PIWI) of *Eh*Ago2-2 with two PIWI proteins in higher eukaryotes (*Hs*HIWI [33563234] and *Dm*PIWI [17136736]) are drawn to scale for comparison, showing that *Eh*Ago2-2 has an extended 200-aa region after PIWI. The C terminus of *Eh*Ago2-2 consists of a possible RGG/RG motif (green) and 12 repetitive RRDDR sequences that range from 8 to 14 aa. The dashed box lists the repeat sequences of C-terminal *Eh*Ago2-2, arranged into separate lines for each RRDDR motif with highlights of arginine (R) (red) and aspartic acid (D) (blue). The underlined sequence is the predicted NLS. Two deletion mutants are made at positions as shown. A chimeric protein, *Eh*Ago2-3 with the DR-rich motif region from *Eh*Ago2-2, is illustrated to scale. (B) Two C-terminal deletion mutants (Myc-*Eh*Ago2-2 DR-deletion and Myc-*Eh*Ago2-2 NLS-DR-deletion) cause Myc-*Eh*Ago2-2 protein localization to the cytosol. Anti-Myc IFAs for both wild type and two deletion mutants are shown. The wild-type parasites have signal in the nucleus, which is in contrast with the deletion mutants, which are largely cytosolic. (C) The *Eh*Ago2-2 DR-rich motif region directs *Eh*Ago2-3 to the nucleus. Anti-Myc IFA shows that the chimeric protein *Eh*Ago2-3 with the DR-rich motif region is localized in the nucleus, compared with its wild-type localization, which is perinuclear and cytoplasmic. (D) sRNA binding ability is unaffected either in the two *Eh*Ago2-2 deletion mutants or in the *Eh*Ago2-3 chimeric protein. The upper panel shows pCp-labeled IP sRNA for all cell lines, indicating that the bound sRNAs are in each line except control. The lower panel shows Western blot analysis for efficiency of Myc pulldown of proteins. With the addition of the DR-motif region tail, the *Eh*Ago2-3 chimeric protein migrates to a higher molecular weight as expected.

An interesting structural difference between *Eh*Agos and model system human HIWI and *Dm*PIWI is the presence in *Eh*Ago2-1 and *Eh*Ago2-2 of extended long tail sequences (∼200 amino acids [aa]) after the PIWI domain (Fig. 4A). While a BLAST search for *Eh*Ago2-1 tail sequences failed to show any meaningful homologous hits, the *Eh*Ago2-2 C-terminal tail sequence is strikingly DR rich and comprises 12 repeats of RRDDR motif sequences (Fig. 4A). Searches using both AmoebaDB and the NCBI BLAST tool found that this structure is not recognized as any known annotated functional domain; instead, it was designated a low-complexity segment. In E. histolytica, a single additional protein with this repeat was found, a U1 snRNA protein with likely nuclear localization (EHI_153670). Performing BLASTP on all eukaryotic genomes, we identified ∼200 hits with ≥5 repeats of the RRDDRD motif (Table S3). Interestingly, most of these hits are from nonmodel systems, and Gene Ontology (GO) analysis indicated that many are either nuclear or RNA binding (Table S2). Out of all published Agos, there is only one hypothetical Piwi protein (EGT41773) from Caenorhabditis brenneri, with the DR-rich motif stretch appearing before the Piwi domain (Table S3).

We further used several online NLS prediction programs (see Materials and Methods) and found a weak NLS sequence (GGRGFGGG) at the *Eh*Ago2-2 C terminus, which is embedded within a stretch of sequences with a possible RGG/RG motif (Fig. 4A). This RGG/RG motif involves protein methylation at arginine (R), which is important for protein functioning in signaling, sorting, and transport and transcription activation (43). In order to study the function of this putative NLS and the DR-rich motif, we generated two deletion mutants: *Eh*Ago2-2 DR-deletion and *Eh*Ago2-2 NLS-DR-deletion (Fig. 4A). We overexpressed Myc-tagged protein for both deletion cell lines and used Western blotting to detect the predicted protein size (Fig. S5). Both deletion mutant proteins of *Eh*Ago2-2 localized to the cytoplasm (Fig. 4B). IFAs using anti-*Eh*Ago2-2 antibody showed that localization of endogenous *Eh*Ago2-2 is unaffected (Fig. S6). As the *Eh*Ago2-2 DR-deletion cell line still contains the predicted NLS sequence, it indicates that deletion of the DR-rich motif itself is sufficient to direct the change of *Eh*Ago2-2 localization and does not require the predicted weak NLS sequence.

10.1128/mSphere.00580-19.5 FIG S5 Expression of *Eh*Ago2-2 DR-deletion and NLS-DR-deletion mutants. Lysates were made from both wild-type and overexpressing Myc-tagged cell lines of Myc-*Eh*Ago2-2 and its DR-deletion and NLS-DR-deletion mutants. Anti-Myc Western blotting detects the expected protein size of both wild-type and deletion proteins (upper panel). The endogenous *Eh*Ago2-2 protein level is checked by using anti-*Eh*Ago2-2 antibody after stripping the membrane (lower panel). Download FIG S5, PDF file, 0.3 MB. Copyright © 2019 Zhang et al. 2019 Zhang et al. This content is distributed under the terms of the Creative Commons Attribution 4.0 International license.

10.1128/mSphere.00580-19.6 FIG S6 The localization of endogenous *Eh*Ago2-2 is unchanged in the overexpressing cell lines of two C-terminal deletion mutants (Myc-*Eh*Ago2-2 DR-deletion and Myc-*Eh*Ago2-2 NLS-DR-deletion). Anti-*Eh*Ago2-2 IFAs for both wild type and two deletion mutants are shown. The nuclear signal is seen in all three cell lines; however, significant cytoplasmic signal is seen for the two deletion mutants due to the fact that the antibody recognizes the Myc-tagged deletion mutant proteins. Download FIG S6, PDF file, 1.0 MB. Copyright © 2019 Zhang et al. 2019 Zhang et al. This content is distributed under the terms of the Creative Commons Attribution 4.0 International license.

We reasoned that *Eh*Ago2-2 could use its DR-rich tail region for nuclear localization. In order to test this idea, we made a chimeric *Eh*Ago2-3 protein with the tail region from *Eh*Ago2-2 fused to its C terminus (Fig. 4A), thus achieving a similar layout structure as in *Eh*Ago2-2. IFA shows that the *Eh*Ago2-3 chimeric protein is now localized to the entire nucleus (instead of its wild-type location in the perinuclear and cytosolic region) (Fig. 4C). This nuclear localization is similar to the localization for *Eh*Ago2-2, indicating that the fused tail region of *Eh*Ago2-2 is sufficient to localize another Ago protein to the nucleus. Last, we checked sRNA binding using anti-Myc IP for these deletion mutants and the chimeric protein of *Eh*Ago2-3. The data show that both *Eh*Ago2-2 deletion mutant proteins bind 27-nt sRNA comparably to the wild-type *Eh*Ago2-2 (Fig. 4D). However, *Eh*Ago2-3 chimeric protein shows a slightly different pattern; the 20- to 24-nt smear sRNA is no longer as obvious as its wild-type counterpart, possibly due to an exonuclease environment change of its changed localization.

In *Entamoeba*, molecular determinants for the protein trafficking into the nucleus are not well studied (44). The typical classical nuclear localization sequences (NLS of the simian virus 40 [SV40] large T antigen) do not appear to work for amebic parasites (reference 44 and data not shown). The previously described NLS from an E. histolytica protein, *Eh*NCABP166 (45), did not work for several proteins in our experience (data not shown). Our data on the *Eh*Ago2-2 tail region and its resulting NLS effect indicate that nuclear transport pathways are active in E. histolytica and that small regulatory RNAs and RISC are transported to the nucleus.

### *Eh*Ago2-2 PIWI mutants have no effect on protein localization and sRNA binding.

The PIWI domain folds like RNase H and has endonucleolytic activity in some Agos for target mRNA cleavage (39). The crystal structure of Ago proteins further reveals a D-D-X triad motif (where X is D/E/H/K) for Ago-based Slicer. However, the *Eh*Ago PIWI domain sequences appear to be very divergent, and the alignment of *Eh*Ago PIWI domain with model systems shows that none have a complete D-D-X catalytic triad (Fig. S7), suggesting that all three *Eh*Agos are likely non-Slicer Agos. The only partially aligned triad position for *Eh*Ago2-2 is D-G-N, which we analyzed by mutagenesis as indicated in Fig. 1.

10.1128/mSphere.00580-19.7 FIG S7 Three *Eh*Ago PIWI domains appear to not bear the D-D-X triad motif. The PIWI domain sequences of three *Eh*Ago proteins along with PIWI sequences from *Hs*HIWI and *Dm*PIWI are aligned using Clustal alignment (www.ebi.ac.uk/Tools/msa/clustalo/). *Eh*Ago PIWI domain sequences appear to be very divergent, and the alignment of *Eh*Ago PIWI domains with model systems shows that none has a complete D-D-X catalytic triad as indicated by blue boxes. *Eh*Ago2-2 PIWI shows a partial alignment as D-G-N. The RNase H-like D-D-E motif is indicated by a red box, showing that the glutamate (E) position is conserved. The positions of PIWIs are as follows: *Eh*Ago2-1, 451 to 755; *Eh*Ago2-2, 467 to 758; *Eh*Ago2-3, 438 to 710; *Hs*HIWI, 555 to 847; *Dm*PIWI, 538 to 829. Download FIG S7, PDF file, 0.2 MB. Copyright © 2019 Zhang et al. 2019 Zhang et al. This content is distributed under the terms of the Creative Commons Attribution 4.0 International license.

**FIG 5:**
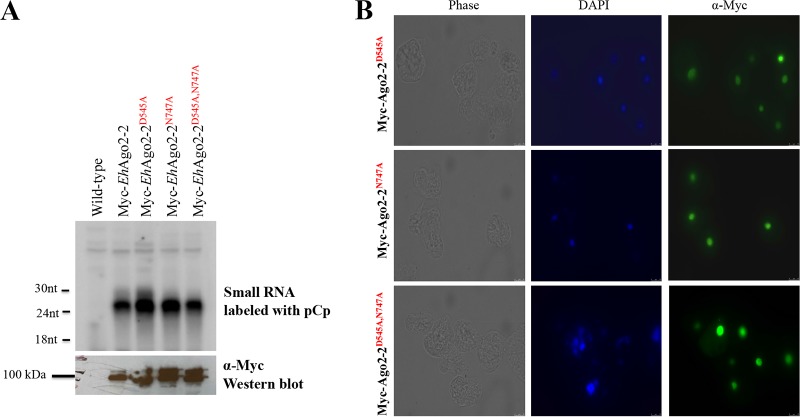
*Eh*Ago2-2 PIWI mutants have no effect on sRNA binding or protein localization. (A) sRNA binding ability is unaffected in three PIWI mutants. Overexpressing Myc-tagged cell lines of three PIWI mutants (*Eh*Ago2-2 PIWI^D545A^, *Eh*Ago2-2 PIWI^N747A^, and *Eh*Ago2-2 PIWI^D545A;N747A^) were used for anti-Myc IP pulldown experiments. The upper panel shows pCp-labeled IP sRNA for all cell lines, indicating that the bound sRNAs are unchanged for PIWI mutants. The lower panel shows anti-Myc Western blot analysis for the IP proteins. (B) *Eh*Ago2-2 localization is unchanged in the overexpressing cell lines of three PIWI mutants. Anti-Myc IFAs for all three PIWI mutants show signal in the nucleus.

We generated three cell lines, *Eh*Ago2-2 PIWI^D545A^, *Eh*Ago2-2 PIWI^N747A^, and *Eh*Ago2-2 PIWI^D545A;N747A^. All Myc-tagged mutants were checked by Western blotting for expression, and the sRNA binding profile was detected by radioactive pCp labeling of RNA prepared from anti-Myc IP samples. Analysis of Ago-bound sRNAs revealed that the 27-nt sRNA population is associated with both the wild type and PIWI mutants, indicating that sRNA binding is intact in these mutants (Fig. 5A). IFAs of these mutants using anti-Myc antibody further showed almost exclusively nuclear localization, similar to the wild-type parasites (Fig. 5B). These results indicate that *Eh*Ago2-2 appears to be tolerant in terms of sRNA binding and localization on its PIWI mutants, implying that *Eh*Ago2-2 may be indeed a non-Slicer.

## DISCUSSION

The RNAi pathway regulates gene expression and is mediated by Ago and its bound small RNAs. Here, we report functional characterization of three Ago proteins in E. histolytica, *Eh*Ago2-1, *Eh*Ago2-2, and *Eh*Ago2-3. We have defined their subcellular localization and showed how environmental stress impacts their localization. Mutagenesis analyses of the *Eh*Ago proteins demonstrated that the PAZ domain is essential for *Eh*Ago sRNA binding, indicating a conserved role of the PAZ domain in sRNA loading in this organism. We further dissected the functional role of an *Eh*Ago2-2 repetitive DR-rich motif and found that this region can function as a nuclear localization signal and is sufficient to mediate nuclear localization of another Ago protein (*Eh*Ago2-3). The DR-rich motif region in *Eh*Ago2-2 has not previously been defined in other systems and adds to the novel observations that can be made when studies of the RNAi pathway are extended to nonmodel systems.

Our data revealed that *Eh*Ago proteins have distinct localizations: the nucleus (*Eh*Ago2-2), perinuclear ring (*Eh*Ago2-1 and *Eh*Ago2-3), cytosol (*Eh*Ago2-3), and cell surface membrane (*Eh*Ago2-1). The nucleus and perinuclear ring localizations are indicative of cellular locations for the TGS pathway that we and the others reported earlier in this parasite (14, 46–48). Whether or not cytoplasmic *Eh*Ago2-3 has a functional role in the PTGS process in this organism is currently unknown. The apparent cell surface membrane localization of *Eh*Ago2-1 is quite interesting, as some Agos in model systems have been reported to be present in both soluble and membrane fractions. Membrane-bound Ago proteins are often found either in the vicinity of the Golgi complex and endoplasmic reticulum or at endosomes/multivesicular bodies (MVBs), and their functions are thought to be related to sRNA loading or RISC assembly (49). Recently, studies on exosomes have shown that they contain cargos including Ago and a specific subset of miRNAs and mediate cell-to-cell communication or host-pathogen interaction (50). Thus, localization of *Eh*Ago to the membrane could indicate that this parasite can use a similar strategy to assemble RISC or to release exosomes to deliver nucleic acid to surrounding parasites or to host cells. Future efforts will be important to pursue this avenue of investigation.

Our data also indicated that three *Eh*Agos undergo localization changes in response to stress conditions. E. histolytica survives under harsh environmental conditions as well as inside host tissues, and previous studies revealed genome-wide gene regulation changes under stress conditions (35, 37, 51). In E. histolytica, two cytoplasmic granules, P‐bodies and stress granule-like structures, have been reported (33, 34), and these organelles have previously been shown as important sites for some RNAi pathways in model systems (2). We speculate that the parasite may use a dynamic network of *Eh*Ago complexes, which vary in composition and localization at distinct cellular sites of action under different cellular stimuli. Further determination of how the induction of cell stress can lead to altered Ago-containing complexes and the biological consequences of these changes is needed.

Our mutagenesis analysis results are summarized in Table 1. For *Eh*Ago PAZ domains, all three *Eh*Ago proteins abolished/severely degraded sRNA binding upon mutations on the conserved residues in the PAZ domain, confirming a conserved role of binding the 3′ end of RNA among all Ago proteins (19). The PAZ mutants also altered the perinuclear ring localization for *Eh*Ago2-1 and *Eh*Ago2-3 but did not alter *Eh*Ago2-2 nuclear localization. We have also identified an unusual DR-rich motif in *Eh*Ago2-2 which appears to control the localization of this protein to the nucleus; two deletion mutants indicated that the DR-rich motif does not alter sRNA binding but is sufficient to mediate nuclear localization of another *Eh*Ago protein. These findings are suggestive that this DR-rich motif may serve as an NLS. For *Eh*Ago PIWI domains, PIWI mutants of *Eh*Ago2-2 did not affect sRNA binding or localization; thus, the exact role of the PIWI domain of *Eh*Agos needs further future study. Future studies using these mutants for an *in vitro* Slicer activity assay will help to elucidate specific PIWI domain function and determine if any of the *Eh*Agos contain Slicer function in this organism.

**TABLE 1:** Summary of all mutant overexpression *Eh*Ago cell lines used in this study and effect on protein localization and sRNA binding^*a*^

Cell line	Mutation(s)	Protein localization	sRNA binding
Wild type			
Myc-*Eh*Ago2-1	None	Cytoplasm/perinuclear ring	Yes
Myc-*Eh*Ago2-2	None	Nucleus	Yes
Myc-*Eh*Ago2-3	None	Cytoplasm/perinuclear ring	Yes
PAZ mutants			
Myc-*Eh*Ago2-1^PAZ-mut^	Y267A, Y268A	Cytoplasm	No
DHFR-Myc-*Eh*Ago2-2^PAZ-mut^	Y267A, Y268A	Nucleus	No
Myc-*Eh*Ago2-3^PAZ-mut^	Y259A, Y260A	Cytoplasm	No
*Eh*Ago2-2 C-terminal mutants			
Myc-*Eh*Ago2-2^ΔDR^	783–937 deletion	Cytoplasm	Yes
Myc-*Eh*Ago2-2^ΔNLS-DR^	761–937 deletion	Cytoplasm	Yes
*Eh*Ago2-2 PIWI mutants			
Myc-*Eh*Ago2-2^D545A^	D545A	Nucleus	Yes
Myc-*Eh*Ago2-2^N747A^	N747A	Nucleus	Yes
Myc-*Eh*Ago2-2^D545A;N747A^	D545A, N747A	Nucleus	Yes
Chimeric protein			
Myc-*Eh*Ago2-3 plus C-terminal tail region of *Eh*Ago2-2	Full-length *Eh*Ago2-3 fused with *Eh*Ago2-2 tail (761–937)	Nucleus	Yes

a Listed are cell lines, specific mutations, protein localizations determined by anti-Myc IFA, and sRNA binding ability determined by anti-Myc IPs and pCp labeling of sRNAs.

**FIG 6:**
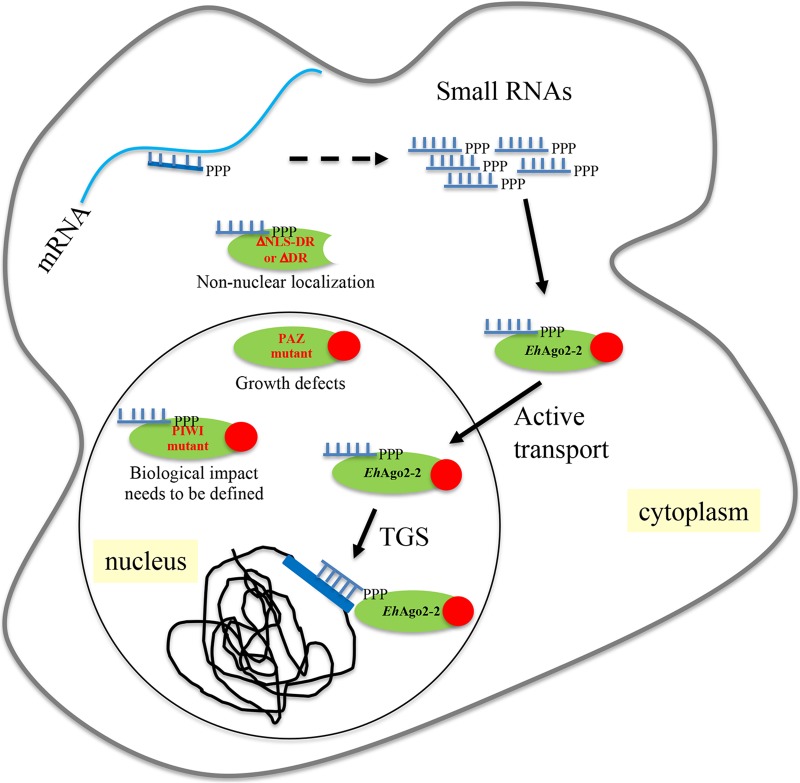
The E. histolytica RNAi pathway involves *Eh*Ago2-2 and its nuclear transport process. In this model, the polyP 27-nt sRNAs are probably generated using low levels of mRNA as the template (dashed arrow). *Eh*Ago2-2 is illustrated as a green oval with its DR-motif region shown as a solid red circle. After loading of 27-nt sRNA into *Eh*Ago2-2, the RISC is translocated into the nucleus using the DR-rich tail region as an NLS. Within the nucleus, the specific locus for TGS is determined by recognition of RISC and its guide sRNAs, which leads to locus-specific histone H3 modification (H3K27Me2 marker) for long-term silencing. The three mutants (ΔNLS-DR or ΔDR, PAZ mutant, and PIWI mutant) are shown along with their localization, sRNA binding, and functional impact. In this study, we show the localization and effects of the three *Eh*Ago2-2 mutants and conclude that the tail of the DR-motif region is responsible for active nuclear transport, while the PAZ domain provides full protection of integrity of sRNA at the 3′ end.

With the data presented in this paper as well as previous work (13–15, 23), including a strong growth defect shown by a PAZ mutant of *Eh*Ago2-2, a working model for the mechanism of TGS in E. histolytica is proposed (Fig. 6). In this model, the polyP 27-nt sRNAs (probably generated by RdRP using a low level of mRNA as the template) are loaded into *Eh*Ago2-2, which needs the PAZ domain for sRNA 3′-end binding and provides full protection of sRNA. After sRNA loading, the RISC is translocated into the nucleus, using the tail region with the DR-rich motif as an NLS. The RISC targets specific loci for TGS using sRNA as a base-pairing guide, which leads to specific histone H3 modification for long-term silencing; we have previously shown that gene silencing in amebae is linked to a histone H3K27Me2 marker at both episomal and chromosomal loci through *Eh*Ago2-2 (15). A similar pathway was also observed in C. elegans, where the Ago protein (NRDE3) uses a bipartite NLS that actively transports siRNAs from the cytoplasm to the nucleus for targeting nascent RNA transcripts and building repressive chromatin marks (40, 52). At present, we do not know the exact roles of *Eh*Ago2-1 and *Eh*Ago2-3 other than their localization and sRNA binding abilities. What small RNAs they bind and whether they also participate in this working model or possess their own pathways need to be further studied.

Overall, our data provide the first comprehensive functional analyses of the three E. histolytica Ago proteins. Identification of the unique DR-rich domain in *Eh*Ago2-2 that functions as an NLS adds to the mechanisms of protein localization. Future studies on identification of the sRNAs that bind to the three *Eh*Ago proteins and the Ago protein silencing complex components will give more insights on the specific roles of the Ago proteins in amebic biology.

## MATERIALS AND METHODS

### Parasite culture and cell lines.

E. histolytica trophozoites (HM-1:IMSS) were grown axenically under standard conditions (in glass culture tubes with TYI-S-33 medium at 36.5°C) as described previously (13, 53). Three *Eh*Ago cell lines constitutively overexpressing N-terminally Myc-tagged Ago protein were made by using the plasmid pKT3M as a backbone and cloned full-length sequences of the three Ago genes (EHI_186850, EHI_125650, and EHI_177170). The PCR products of three Ago genes were amplified from genomic DNA using primers listed in Table S1 in the supplemental material and cloned with SmaI and XhoI sites. The resulting constructs are pKT3M-Myc-*Eh*Ago2-1, pKT3M-Myc-*Eh*Ago2-2 (reported in reference 13 and used as a control), and pKT3M-Myc-*Eh*Ago2-3. To make *Eh*Ago PAZ and PIWI domain mutants, we used the NEB Gibson assembly cloning kit (New England Biolabs). The two fragments were PCR amplified for each *Eh*Ago using primers listed in Table S1 and assembled with pKT3M-Myc backbone using protocols provided in the kit; the resulting constructs are pKT3M-Myc-*Eh*Ago2-1^PAZ-mut^, pKT3M-Myc-*Eh*Ago2-2^PAZ-mut^, and pKT3M-Myc-*Eh*Ago2-3^PAZ-mut^. To make the *Eh*Ago2-2^PAZ-mut^ destabilization domain construct, we inserted the dihydrofolate reductase (DHFR) gene in-frame at the SmaI site between 3×Myc and *Eh*Ago2-2^PAZ-mut^ based on pKT3M-Myc-*Eh*Ago2-2^PAZ-mut^ plasmid, resulting in pKT3M-Myc-DHFR-*Eh*Ago2-2^PAZ-mut^. To make *Eh*Ago2-2 C-terminal deletion mutant constructs, we used the forward primer of *Eh*Ago2-2 and the reverse primers of del-DR and del-NLS-DR as listed in Table S1; the resulting constructs are pKT3M-Myc-*Eh*Ago2-2 DR-deletion and pKT3M-Myc-*Eh*Ago2-2 NLS-DR-deletion; the primer sets amplify *Eh*Ago2-2 for amino acids 1 to 783 and 1 to 761, respectively. To make *Eh*Ago2-3 plus the DR-rich motif region of *Eh*Ago2-2, we first performed site-directed mutagenesis on pKT3M-Myc-*Eh*Ago2-2 using the QuikChange Lightning kit (Agilent) to generate a unique AvrII site at amino acid position 762 before the DR-rich motif region, and then full-length *Eh*Ago2-3 was subjected to PCR using primers (forward primer of *Eh*Ago2-3 and its AvrII reverse primer). The PCR product was cloned using SmaI and AvrII sites. The three *Eh*Ago2-2 PIWI mutants were also constructed using the NEB Gibson assembly cloning kit (New England Biolabs) using primers listed in Table S1. All plasmid constructs were confirmed by sequencing, and parasite transfectants were maintained at 6 μg/ml G418.

10.1128/mSphere.00580-19.8 TABLE S1 All oligonucleotides used in this study. Download Table S1, PDF file, 0.07 MB. Copyright © 2019 Zhang et al. 2019 Zhang et al. This content is distributed under the terms of the Creative Commons Attribution 4.0 International license.

10.1128/mSphere.00580-19.9 TABLE S2 Pfam domain analysis results for proteins with RRDDRD motif. BLASTP search on all eukaryotic genomes identified ∼200 protein hits with ≥5 repeats of the RRDDRD motif. Pfam domain analysis for these proteins was done using www.ebi.ac.uk/Tools/hmmer/search/phmmer. Listed are most representative Pfam results with ≥3 protein hits. Of note, all PAZ and PIWI proteins are from ameba species except one PIWI protein (EGT41773) from *C. brenneri*. The nuclear localization is indicated by transcription and elongation Pfam proteins (Sin3_corepress, Sin3a_C, THOC2_N, DEAD, and Helicase_C), and RNA interaction is indicated by Pfam proteins (RRM_1, S1, PAZ, and PIWI). Download Table S2, PDF file, 0.06 MB. Copyright © 2019 Zhang et al. 2019 Zhang et al. This content is distributed under the terms of the Creative Commons Attribution 4.0 International license.

10.1128/mSphere.00580-19.10 TABLE S3 RRDDRDR_proteins.txt. The RRDDRDR motif was used to search all eukaryotic proteins with the following parameters: E value, 1,000; word size, 2; window size, 40. Proteins with 5 or more copies of the repeat were identified, and conserved domains were identified (http://pfam.xfam.org/). All the protein sequences identified in this manner are in the table. Download Table S3, TXT file, 0.2 MB. Copyright © 2019 Zhang et al. 2019 Zhang et al. This content is distributed under the terms of the Creative Commons Attribution 4.0 International license.

### Polyclonal antibody production and immunofluorescence assays.

*Eh*Ago polyclonal antibodies were generated in rabbits. The antigen peptide used for *Eh*Ago2-2 was reported in reference 23. For *Eh*Ago2-1, peptide sequence C-KKPLYDLYTQQYSFITNYFE-NH_2_, corresponding to the N-terminal amino acids 31 to 50 of *Eh*Ago2-1, was used. For *Eh*Ago2-3, peptide sequence C-DDPYPKEFTDLIPQYEDVDD-NH_2_, corresponding to the N-terminal amino acids 25 to 44 of *Eh*Ago2-3, was used. The antibodies were affinity purified on a peptide column (Open Biosystems). For IFA, we fixed cells using methanol-acetone (1:1) and followed previously reported protocols (23). Primary antibody (custom-made polyclonal antibodies for three *Eh*Agos and anti-*Eh*fibrillarin [27] [a gift from Sudha Bhattacharya] or commercial anti-Myc) was used at a 1:250 dilution and incubated at 4°C overnight. Alexa 488 (anti-rabbit or anti-mouse) (Molecular Probes) was used as secondary antibody at a 1:1,000 dilution. Slides were mounted with Vectashield mounting medium (Vector Laboratories, Inc.), and images were collected using a Leica CTR6000 microscope using a BD CARVII confocal unit.

### Stress stimuli and conditions.

Both stress conditions were the same as previously published, H_2_O_2_ stress (1 mM H_2_O_2_ for 1 h) (35) and heat shock (42°C for 1 h) (36). We used E. histolytica HM-1:IMSS culture tubes for stress treatments. After treatment, tubes were shaken gently on the rim of an ice bucket to release any remaining attached cells, and the cells were collected by spinning at 1,000 rpm for 5 min and washed once with 1× phosphate-buffered saline (PBS). The cells were then transferred to Eppendorf tubes for methanol-acetone (1:1) fixation, followed by incubation of primary and secondary antibodies as described above; after a final wash, cells were mounted on the coverslip for imaging.

### Cell fractionation for cytoplasmic and nuclear lysates.

We followed the same protocol used in the lab (37). Briefly, both untreated and stress-treated cells were collected; resuspended in buffer A (10 mM HEPES, pH 7.9, 1.5 mM MgCl_2_, 10 mM KCl, and 0.6% Igepal) with protease inhibitors, 1 μm leupeptin, 1 μm E-64-d, and 1× HALT protease inhibitor mixture (Pierce); and incubated on ice for 20 min. After low-speed centrifugation for 10 min at 1,000 × *g* at 4°C, the supernatant was collected as cytoplasmic fraction lysate. The pellet was then washed and resuspended in buffer C (20 mM HEPES, pH 7.9, 420 mM NaCl, 1 mM EDTA, and 1 mM EGTA supplemented with the same protease inhibitor mixture) on ice for 30 min. The supernatant from the last centrifugation at 18,000 × *g* for 20 min was collected as nuclear fraction lysate.

### Cell lysate, immunoprecipitation (IP), and RNA isolation.

We used a protocol similar to that in reference 54 with modifications. The lysis buffer (20 mM Tris-HCl [pH 7.5], 1 mM MgCl_2_, 10% [vol/vol] glycerol, and 50 mM NaCl) contained 0.5% (vol/vol) Nonidet P-40 (NP-40), plus 1 mM NaF, 1 mM dithiothreitol (DTT), 1 mM phenylmethylsulfonyl fluoride (PMSF), 2× of 100× Halt EDTA-free protease inhibitors (100× stock diluted to 2× as final concentration; Thermo Scientific), and 1× RNase inhibitor, 1 U/ml. The cells from one T25 flask were lysed in 600 μl of lysis buffer and incubated on ice for 15 min. Lysate was centrifuged at 14,000 rpm for 30 min in a benchtop centrifuge at 4°C, and supernatant was saved at −80°C.

For IP with anti-Myc beads (Thermo Scientific), 20 μl packed beads was rinsed twice with cold 1× Dulbecco PBS (DPBS) with 0.01% Tween 20. After washing, 500 μl whole-cell lysate (1 to 2 μg/μl) was added and incubated for 2 h with rotation at 4°C, and the beads were then pelleted and washed six times (5 min each) under low-stringency conditions in basic lysis buffer containing PMSF, 0.1% (vol/vol) Tween 20, and 0.1% (vol/vol) NP-40 at 4°C. We collected both IP RNA and IP protein samples for each IP sample by splitting the bead-wash mixture at the last wash step. The IP RNA was isolated using TRIzol (Invitrogen) reagent according to standard protocol, and the IP proteins were released by adding 30 μl 1× reducing lane marker buffer (Thermo Scientific) and heated at 95°C for 5 min.

### RNA pCp labeling.

We followed similar procedures as in reference 13 for RNA labeling at 3′ termini. Typically, 2 μl RNA was ligated to [α-^32^P]pCp using T4 RNA ligase (New England Biolabs), and the reaction mixture was incubated at room temperature for 2 h. The ligation reaction mixture was resolved directly on a denaturing 7 M urea-containing 15% polyacrylamide gel, and signal was exposed to a phosphor screen and imaged on a Personal molecular imager (Bio-Rad).

### Western blot analysis.

Standard Western blotting technique was used. We used 5% milk as a blocking reagent, and primary antibody was used at a 1:1,000 dilution, with incubation at 4°C overnight. The appropriate secondary antibody was used at a 1:10,000 dilution and incubated at room temperature for 1 h. Signal was developed with ECL+ (GE Healthcare, USA) and detected by the Kodak Image Station 4000R (Kodak, USA) or with a film processor (GE Healthcare). Antibodies used in Western blot analysis were anti-Myc antibody (Cell Signaling), anti-actin antibody (Cell Signaling), and the custom-made *Eh*Ago polyclonal antibodies.

### Protein stabilization conditions and growth kinetics.

For Western blot analysis, trophozoites expressing Myc-DHFR-*Eh*Ago2-2^PAZ-mut^ were collected after adding stabilization compound (10 μM TMP) for 24 h, and protein lysates were made and analyzed by Western blotting using anti-Myc antibody (Cell Signaling). For determination of growth kinetics, parasites were seeded at 40,000 cells/tube, no-drug control along with stabilizing compound (10 μM TMP) was set up in duplicate, and the number of parasites was counted every 24 h for 5 consecutive days after exposure to stabilizing compound.

### Sequence alignment, phylogenetic analysis, and motif search.

The three *Eh*Ago sequences and their annotations, including Superfamily and Pfam domain regions, are based on www.amoebadb.org/amoeba. To search for orthologs in other ameba species, we used the orthology and synteny functional tab that is a built-in function for each gene in AmoebaDB. We then used full sequences to build a phylogenetic tree using a web tool (www.phylogeny.fr/). The Clustal Omega tool was used for PAZ and PIWI alignment analysis (www.ebi.ac.uk/Tools/msa/clustalo/). For NLS prediction, we used several online tools, including NLS mapper (http://nls-mapper.iab.keio.ac.jp/cgi-bin/NLS_Mapper_form.cgi), PredictProtein (www.predictprotein.org), and NucPred (https://nucpred.bioinfo.se/nucpred/). For motif search, we used the built-in motif search function in AmoebaDB and NCBI BLASTP. The RRDDRDR motif was used to search all eukaryotic proteins with the following parameters: E value, 1,000; word size, 2; window size, 40. Proteins with 5 or more copies of the repeat were identified, and conserved domains were identified (http://pfam.xfam.org).

## References

[1] Hauptmann J, Meister G 2013 Argonaute regulation: two roads to the same destination. Dev Cell 25:553–554. doi:10.1016/j.devcel.2013.06.009. 23806615

[2] Meister G 2013 Argonaute proteins: functional insights and emerging roles. Nat Rev Genet 14:447–459. doi:10.1038/nrg3462. 23732335

[3] Kuhn CD, Joshua-Tor L 2013 Eukaryotic Argonautes come into focus. Trends Biochem Sci 38:263–271. doi:10.1016/j.tibs.2013.02.008. 23541793

[4] Wilson RC, Doudna JA 2013 Molecular mechanisms of RNA interference. Annu Rev Biophys 42:217–239. doi:10.1146/annurev-biophys-083012-130404. 23654304 PMC5895182

[5] Ghildiyal M, Zamore PD 2009 Small silencing RNAs: an expanding universe. Nat Rev Genet 10:94–108. doi:10.1038/nrg2504. 19148191 PMC2724769

[6] Batista TM, Marques JT 2011 RNAi pathways in parasitic protists and worms. J Proteomics 74:1504–1514. doi:10.1016/j.jprot.2011.02.032. 21385631

[7] Drinnenberg IA, Weinberg DE, Xie KT, Mower JP, Wolfe KH, Fink GR, Bartel DP 2009 RNAi in budding yeast. Science 326:544–550. doi:10.1126/science.1176945. 19745116 PMC3786161

[8] Ullu E, Tschudi C, Chakraborty T 2004 RNA interference in protozoan parasites. Cell Microbiol 6:509–519. doi:10.1111/j.1462-5822.2004.00399.x. 15104593

[9] Haque R, Mondal D, Kirkpatrick BD, Akther S, Farr BM, Sack RB, Petri WA Jr 2003 Epidemiologic and clinical characteristics of acute diarrhea with emphasis on Entamoeba histolytica infections in preschool children in an urban slum of Dhaka, Bangladesh. Am J Trop Med Hyg 69:398–405. doi:10.4269/ajtmh.2003.69.398. 14640500

[10] Stanley SL Jr 2003 Amoebiasis. Lancet 361:1025–1034. doi:10.1016/S0140-6736(03)12830-9. 12660071

[11] Pompey JM, Foda B, Singh U 2015 A single RNaseIII domain protein from Entamoeba histolytica has dsRNA cleavage activity and can help mediate RNAi gene silencing in a heterologous system. PLoS One 10:e0133740. doi:10.1371/journal.pone.0133740. 26230096 PMC4521922

[12] Yu X, Li X, Zheng L, Ma J, Gan J 2017 Structural and functional studies of a noncanonical Dicer from Entamoeba histolytica. Sci Rep 7:44832. doi:10.1038/srep44832. 28317870 PMC5357909

[13] Zhang H, Ehrenkaufer GM, Pompey JM, Hackney JA, Singh U 2008 Small RNAs with 5’-polyphosphate termini associate with a Piwi-related protein and regulate gene expression in the single-celled eukaryote Entamoeba histolytica. PLoS Pathog 4:e1000219. doi:10.1371/journal.ppat.1000219. 19043551 PMC2582682

[14] Morf L, Pearson RJ, Wang AS, Singh U 2013 Robust gene silencing mediated by antisense small RNAs in the pathogenic protist Entamoeba histolytica. Nucleic Acids Res 41:9424–9437. doi:10.1093/nar/gkt717. 23935116 PMC3814356

[15] Foda BM, Singh U 2015 Dimethylated H3K27 is a repressive epigenetic histone mark in the protist Entamoeba histolytica and is significantly enriched in genes silenced via the RNAi pathway. J Biol Chem 290:21114–21130. doi:10.1074/jbc.M115.647263. 26149683 PMC4543668

[16] Zhang H, Ehrenkaufer GM, Hall N, Singh U 2013 Small RNA pyrosequencing in the protozoan parasite Entamoeba histolytica reveals strain-specific small RNAs that target virulence genes. BMC Genomics 14:53. doi:10.1186/1471-2164-14-53. 23347563 PMC3610107

[17] Zhang H, Ehrenkaufer GM, Manna D, Hall N, Singh U 2015 High throughput sequencing of Entamoeba 27nt small RNA population reveals role in permanent gene silencing but no effect on regulating gene expression changes during stage conversion, oxidative, or heat shock stress. PLoS One 10:e0134481. doi:10.1371/journal.pone.0134481. 26248204 PMC4527709

[18] Pompey JM, Morf L, Singh U 2014 RNAi pathway genes are resistant to small RNA mediated gene silencing in the protozoan parasite Entamoeba histolytica. PLoS One 9:e106477. doi:10.1371/journal.pone.0106477. 25198343 PMC4157801

[19] Swarts DC, Makarova K, Wang Y, Nakanishi K, Ketting RF, Koonin EV, Patel DJ, van der Oost J 2014 The evolutionary journey of Argonaute proteins. Nat Struct Mol Biol 21:743–753. doi:10.1038/nsmb.2879. 25192263 PMC4691850

[20] Hock J, Meister G 2008 The Argonaute protein family. Genome Biol 9:210. doi:10.1186/gb-2008-9-2-210. 18304383 PMC2374724

[21] Zhang H, Pompey JM, Singh U 2011 RNA interference in Entamoeba histolytica: implications for parasite biology and gene silencing. Future Microbiol 6:103–117. doi:10.2217/fmb.10.154. 21162639 PMC3038252

[22] Kolev NG, Tschudi C, Ullu E 2011 RNA interference in protozoan parasites: achievements and challenges. Eukaryot Cell 10:1156–1163. doi:10.1128/EC.05114-11. 21764910 PMC3187059

[23] Zhang H, Alramini H, Tran V, Singh U 2011 Nucleus-localized antisense small RNAs with 5’-polyphosphate termini regulate long term transcriptional gene silencing in Entamoeba histolytica G3 strain. J Biol Chem 286:44467–44479. doi:10.1074/jbc.M111.278184. 22049083 PMC3247957

[24] Ehrenkaufer GM, Haque R, Hackney JA, Eichinger DJ, Singh U 2007 Identification of developmentally regulated genes in Entamoeba histolytica: insights into mechanisms of stage conversion in a protozoan parasite. Cell Microbiol 9:1426. doi:10.1111/j.1462-5822.2006.00882.x. 17250591

[25] Gilchrist CA, Houpt E, Trapaidze N, Fei Z, Crasta O, Asgharpour A, Evans C, Martino-Catt S, Baba DJ, Stroup S, Hamano S, Ehrenkaufer G, Okada M, Singh U, Nozaki T, Mann BJ, Petri WA Jr 2006 Impact of intestinal colonization and invasion on the Entamoeba histolytica transcriptome. Mol Biochem Parasitol 147:163–176. doi:10.1016/j.molbiopara.2006.02.007. 16569449

[26] Hon CC, Weber C, Sismeiro O, Proux C, Koutero M, Deloger M, Das S, Agrahari M, Dillies MA, Jagla B, Coppee JY, Bhattacharya A, Guillen N 2013 Quantification of stochastic noise of splicing and polyadenylation in Entamoeba histolytica. Nucleic Acids Res 41:1936–1952. doi:10.1093/nar/gks1271. 23258700 PMC3561952

[27] Jhingan GD, Panigrahi SK, Bhattacharya A, Bhattacharya S 2009 The nucleolus in Entamoeba histolytica and Entamoeba invadens is located at the nuclear periphery. Mol Biochem Parasitol 167:72–80. doi:10.1016/j.molbiopara.2009.04.011. 19416742

[28] Shiu PK, Zickler D, Raju NB, Ruprich-Robert G, Metzenberg RL 2006 SAD-2 is required for meiotic silencing by unpaired DNA and perinuclear localization of SAD-1 RNA-directed RNA polymerase. Proc Natl Acad Sci U S A 103:2243–2248. doi:10.1073/pnas.0508896103. 16461906 PMC1413707

[29] Decker LM, Boone EC, Xiao H, Shanker BS, Boone SF, Kingston SL, Lee SA, Hammond TM, Shiu PK 2015 Complex formation of RNA silencing proteins in the perinuclear region of Neurospora crassa. Genetics 199:1017–1021. doi:10.1534/genetics.115.174623. 25644701 PMC4391574

[30] Billi AC, Fischer SE, Kim JK 2014 Endogenous RNAi pathways in *C. elegans*. WormBook doi:10.1895/wormbook.1.170.1. PMC4781133 24816713

[31] Ishidate T, Ozturk AR, Durning DJ, Sharma R, Shen EZ, Chen H, Seth M, Shirayama M, Mello CC 2018 ZNFX-1 functions within perinuclear nuage to balance epigenetic signals. Mol Cell 70:639–649.e6. doi:10.1016/j.molcel.2018.04.009. 29775580 PMC5994929

[32] Detzer A, Engel C, Wunsche W, Sczakiel G 2011 Cell stress is related to re-localization of Argonaute 2 and to decreased RNA interference in human cells. Nucleic Acids Res 39:2727–2741. doi:10.1093/nar/gkq1216. 21148147 PMC3074141

[33] Katz S, Trebicz-Geffen M, Ankri S 2014 Stress granule formation in Entamoeba histolytica: cross-talk between EhMLBP, EhRLE3 reverse transcriptase and polyubiquitinated proteins. Cell Microbiol 16:1211–1223. doi:10.1111/cmi.12273. 24471581

[34] López-Rosas I, Orozco E, Marchat LA, García-Rivera G, Guillen N, Weber C, Carrillo-Tapia E, Hernández de la Cruz O, Pérez-Plasencia C, López-Camarillo C 2012 mRNA decay proteins are targeted to poly(A)+ RNA and dsRNA-containing cytoplasmic foci that resemble P-bodies in Entamoeba histolytica. PLoS One 7:e45966. doi:10.1371/journal.pone.0045966. 23029343 PMC3454373

[35] Hackney JA, Ehrenkaufer GM, Singh U 2007 Identification of putative transcriptional regulatory networks in Entamoeba histolytica using Bayesian inference. Nucleic Acids Res 35:2141–2152. doi:10.1093/nar/gkm028. 17355990 PMC1874630

[36] Vicente JB, Ehrenkaufer GM, Saraiva LM, Teixeira M, Singh U 2009 Entamoeba histolytica modulates a complex repertoire of novel genes in response to oxidative and nitrosative stresses: implications for amebic pathogenesis. Cell Microbiol 11:51–69. doi:10.1111/j.1462-5822.2008.01236.x. 18778413 PMC3418052

[37] Pearson RJ, Morf L, Singh U 2013 Regulation of H2O2 stress-responsive genes through a novel transcription factor in the protozoan pathogen Entamoeba histolytica. J Biol Chem 288:4462–4474. doi:10.1074/jbc.M112.423467. 23250742 PMC3567695

[38] Ketting RF 2011 The many faces of RNAi. Dev Cell 20:148–161. doi:10.1016/j.devcel.2011.01.012. 21316584

[39] Tolia NH, Joshua-Tor L 2007 Slicer and the argonautes. Nat Chem Biol 3:36–43. doi:10.1038/nchembio848. 17173028

[40] Guang S, Bochner AF, Pavelec DM, Burkhart KB, Harding S, Lachowiec J, Kennedy S 2008 An Argonaute transports siRNAs from the cytoplasm to the nucleus. Science 321:537–541. doi:10.1126/science.1157647. 18653886 PMC2771369

[41] Liu YC, Singh U 2014 Destabilization domain approach adapted for regulated protein expression in the protozoan parasite Entamoeba histolytica. Int J Parasitol 44:729–735. doi:10.1016/j.ijpara.2014.05.002. 24929134 PMC4138259

[42] Ma JB, Ye K, Patel DJ 2004 Structural basis for overhang-specific small interfering RNA recognition by the PAZ domain. Nature 429:318–322. doi:10.1038/nature02519. 15152257 PMC4700412

[43] Thandapani P, O’Connor TR, Bailey TL, Richard S 2013 Defining the RGG/RG motif. Mol Cell 50:613–623. doi:10.1016/j.molcel.2013.05.021. 23746349

[44] Gwairgi MA, Ghildyal R 2018 Nuclear transport in Entamoeba histolytica: knowledge gap and therapeutic potential. Parasitology 145:1378–1387. doi:10.1017/S0031182018000252. 29565001

[45] Uribe R, Almaraz Barrera MDJ, Robles-Flores M, Mendoza Hernández G, González-Robles A, Hernández-Rivas R, Guillen N, Vargas M 2012 A functional study of nucleocytoplasmic transport signals of the EhNCABP166 protein from Entamoeba histolytica. Parasitology 139:1697–1710. doi:10.1017/S0031182012001199. 22906852

[46] Huguenin M, Bracha R, Chookajorn T, Mirelman D 2010 Epigenetic transcriptional gene silencing in Entamoeba histolytica: insight into histone and chromatin modifications. Parasitology 137:619–627. doi:10.1017/S0031182009991363. 19849886

[47] Bracha R, Nuchamowitz Y, Mirelman D 2003 Transcriptional silencing of an amoebapore gene in Entamoeba histolytica: molecular analysis and effect on pathogenicity. Eukaryot Cell 2:295–305. doi:10.1128/ec.2.2.295-305.2003. 12684379 PMC154849

[48] Khalil MI, Foda BM, Suresh S, Singh U 2016 Technical advances in trigger-induced RNA interference gene silencing in the parasite Entamoeba histolytica. Int J Parasitol 46:205–212. doi:10.1016/j.ijpara.2015.11.004. 26747561 PMC4767557

[49] Gibbings DJ, Ciaudo C, Erhardt M, Voinnet O 2009 Multivesicular bodies associate with components of miRNA effector complexes and modulate miRNA activity. Nat Cell Biol 11:1143–1149. doi:10.1038/ncb1929. 19684575

[50] Penfornis P, Vallabhaneni KC, Whitt J, Pochampally R 2016 Extracellular vesicles as carriers of microRNA, proteins and lipids in tumor microenvironment. Int J Cancer 138:14–21. doi:10.1002/ijc.29417. 25559768 PMC4924539

[51] Naiyer S, Kaur D, Ahamad J, Singh SS, Singh YP, Thakur V, Bhattacharya A, Bhattacharya S 2019 Transcriptomic analysis reveals novel downstream regulatory motifs and highly transcribed virulence factor genes of Entamoeba histolytica. BMC Genomics 20:206. doi:10.1186/s12864-019-5570-z. 30866809 PMC6416950

[52] Guang S, Bochner AF, Burkhart KB, Burton N, Pavelec DM, Kennedy S 2010 Small regulatory RNAs inhibit RNA polymerase II during the elongation phase of transcription. Nature 465:1097–1101. doi:10.1038/nature09095. 20543824 PMC2892551

[53] Diamond LS, Harlow DR, Cunnick CC 1978 A new medium for the axenic cultivation of Entamoeba histolytica and other Entamoeba. Trans R Soc Trop Med Hyg 72:431–432. doi:10.1016/0035-9203(78)90144-x. 212851

[54] Lee SR, Collins K 2007 Physical and functional coupling of RNA-dependent RNA polymerase and Dicer in the biogenesis of endogenous siRNAs. Nat Struct Mol Biol 14:604–610. doi:10.1038/nsmb1262. 17603500

