# Supplementary figures and images for: Functional Characterization of Entamoeba histolytica Argonaute Proteins Reveals a Repetitive DR-Rich Motif Region That Controls Nuclear Localization
Source: mSphere. 2019 Oct 16;4(5):e00580-19. doi: 10.1128/mSphere.00580-19 (PMC6796981; doi:10.1128/mSphere.00580-19)

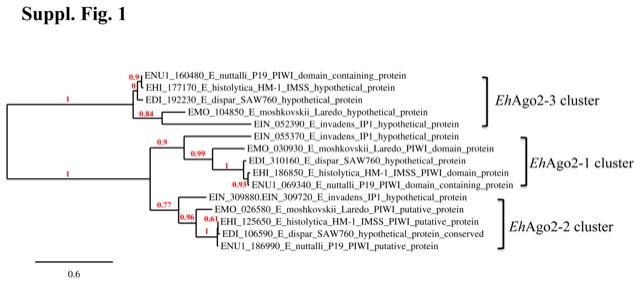

Supplement: FIG S1 [file mSphere.00580-19-sf001.tif]

## Suppl. Fig. 2

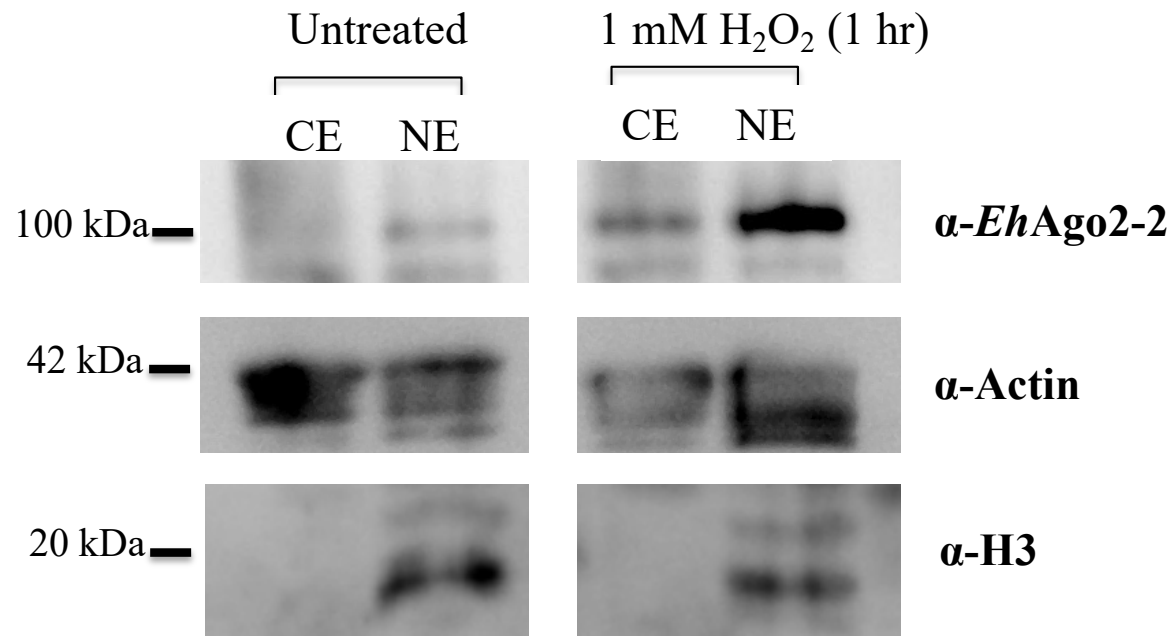

Supplement: FIG S2 [file mSphere.00580-19-sf002.pdf]

## Suppl. Fig. 4

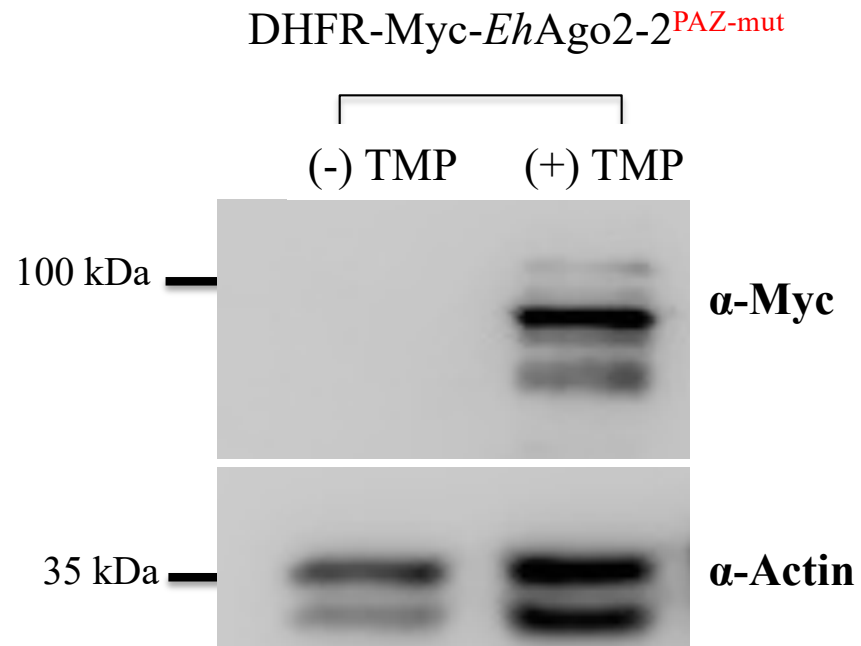

Supplement: FIG S4 [file mSphere.00580-19-sf004.pdf]

## Suppl. Fig. 5

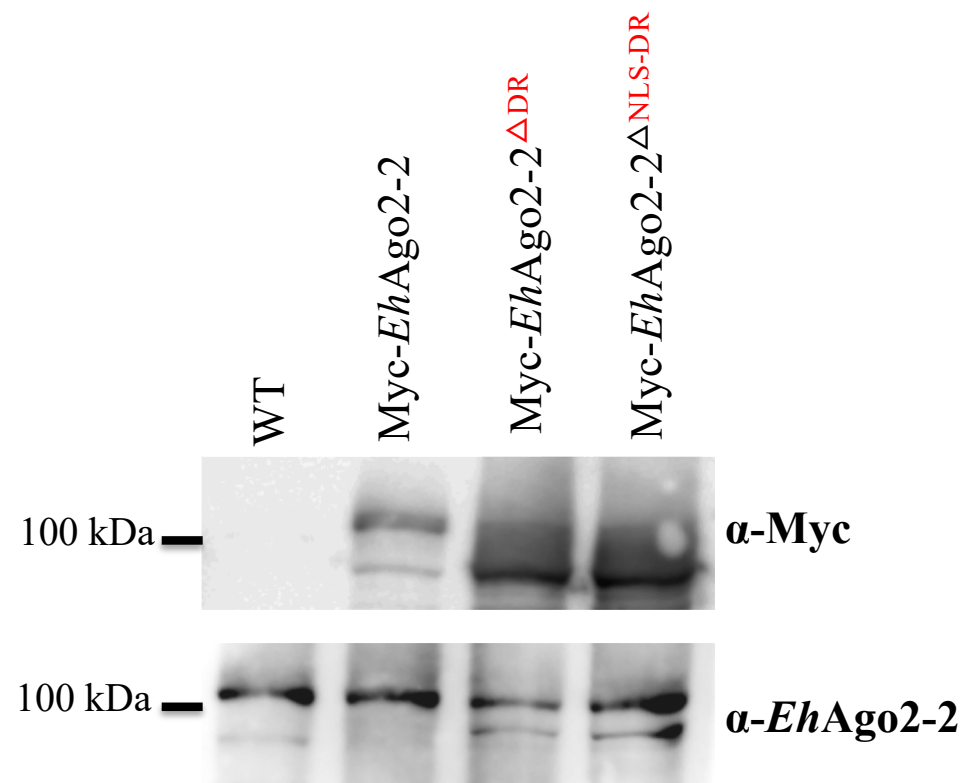

Supplement: FIG S5 [file mSphere.00580-19-sf005.pdf]

Suppl. Fig. 6

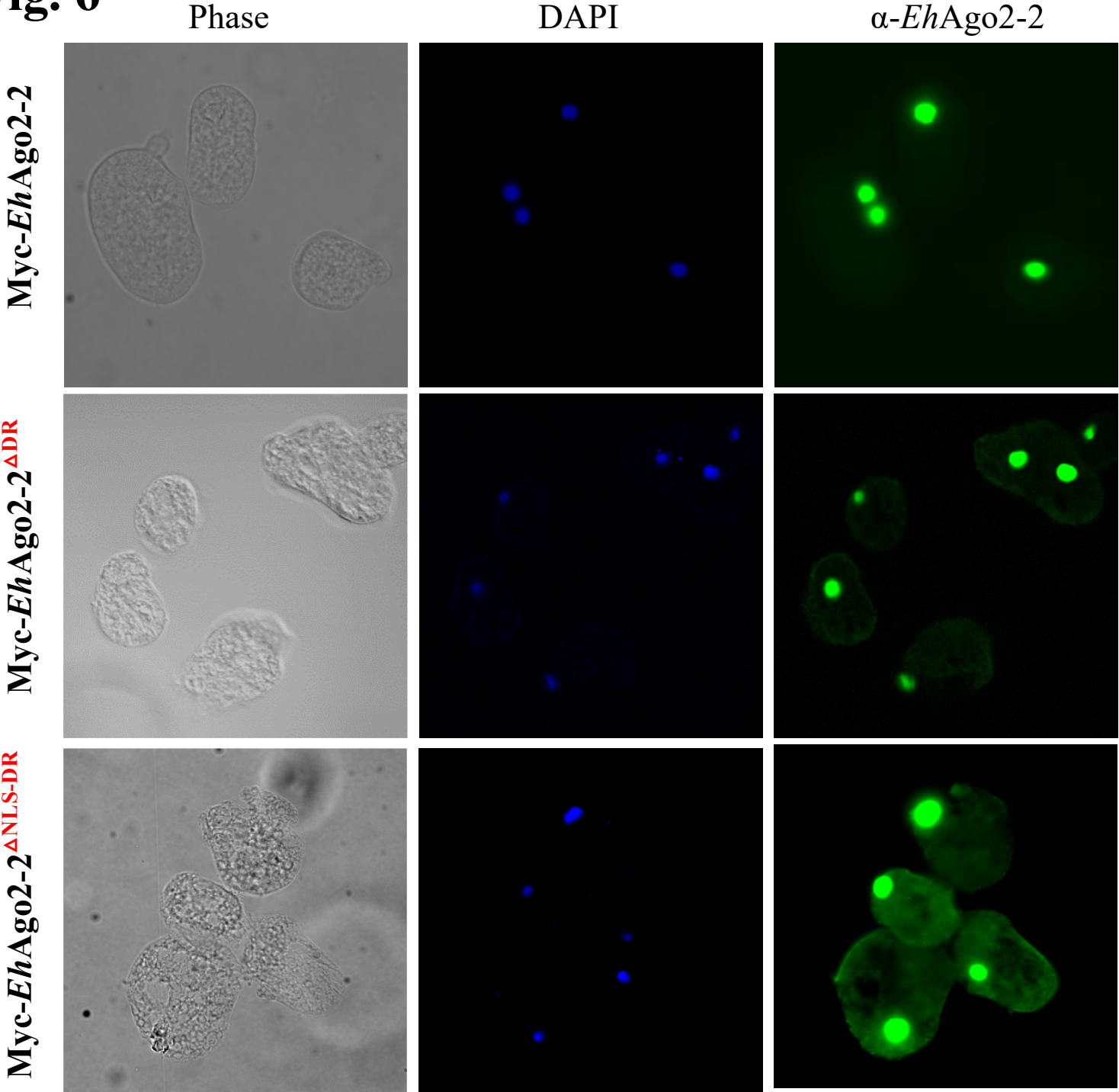

Supplement: FIG S6 [file mSphere.00580-19-sf006.pdf]
