# Supplementary material for: Functional Characterization of Entamoeba histolytica Argonaute Proteins Reveals a Repetitive DR-Rich Motif Region That Controls Nuclear Localization
Source: mSphere. 2019 Oct 16;4(5):e00580-19. doi: 10.1128/mSphere.00580-19 (PMC6796981; doi:10.1128/mSphere.00580-19)
Supplement: FIG S3 [file mSphere.00580-19-sf003.pdf]

Suppl. Fig. 3

```
CLUSTAL O(1.2.4) multiple sequence alignment


Hs_HIWI        ETVLDFMFNFYHQTEEHKFQEQVSKELIGLVVLTKYNNKTYRVDDIDWDQNPKSTFKKAD
Dm_PIWI        ETIYDIMRRCSHNP--ARHQDEVRVNVLDLIVLTDYNNRTYRINDVDFGQTPKSTFS-CK
Eh_Ago2-1      ------------QFMENKGVEQIKFEFKPMSLFNSLTKKLVKIDSIDFSKTPLHTFKLKD
Eh_Ago2-2      ----------YLDSIRSKDQDTIKKEFQPMVLYNSQTKRLEKIDDIDFGKNPSFSFTRKD
Eh_Ago2-3      -----------LEESKEYSYDEIAIKFRPSWYYNEVSKQLEWIDEVKMNVTPDSQKFFWG
                           :       : :  :.      .. .::   ::.:. . .*

Hs_HIWI        GSEVSFLEYYRKQYNQEIT-DLKQPVLVSQPKRRRGPG-GTLPGPAMLIPELCYLTG
Dm_PIWI        GRDISFVEYYLTKYNIRIR-DHNQPLLISKNRDKA-LK-TNASELVVLIPELCRVTG
Eh_Ago2-1      GKEISFIDYYKSKYGVIIK-NTEQPLLVQNNPFFNENN--NSNEFSYFIPEFMYMTG
Eh_Ago2-2      GSSVSIAEYYKQRYQIEIR-DMDQPLLVQKKIEKDSEGKEVEKQPCYFVP-------
Eh_Ago2-3      DQEVSVSQYYMQRYGGAVHFDPNQFLMVQKQRKFG------RIEYAYYVPQLMHRV-
               . .:*. :**  :*   :  : .* :::.:                  :*
```
