# Supplementary material for: Functional Characterization of Entamoeba histolytica Argonaute Proteins Reveals a Repetitive DR-Rich Motif Region That Controls Nuclear Localization
Source: mSphere. 2019 Oct 16;4(5):e00580-19. doi: 10.1128/mSphere.00580-19 (PMC6796981; doi:10.1128/mSphere.00580-19)
Supplement: FIG S7 [file mSphere.00580-19-sf007.pdf]

Suppl. Fig. 7

```
CLUSTAL O(1.2.4) multiple sequence alignment


Hs_Hiwi         ----------------IVVCLLSSNRKDKYDAIKKYLCTDCPTPSQCVVARTLGKQQTVMA
Dm_PIWI         ----------------LILCLVPNDNAERYSSIKKRGYVDRAVPTQVVTLK-TTKNRSLMS
Eh_Ago2-1       MIGLSLDDYFNEILNYKIVFVVILNDSLYEDIK-RKCFDKEIITQCIHPDTL-KKQNLFP
Eh_Ago2-2       ---------------NSSMVLAVSVTDSIYEQIK-KFCVDENVPTQCIKPNTL-RRQPPTP
Eh_Ago2-3       ----------------QIVFSVFDSIEQMVEIKEYFMIYGNIPTQIIDLNERYDDKKITN
                                 : :      .    **          :* :        :

Hs_Hiwi         IATKIALQMNCKMGGELWRVDIPLKL--VMIVGIDCYHDMTAGRRSIAGFVASINEGMT-
Dm_PIWI         IATKIAIQLNCKLGYTPWMIELPLSG--LMTIGFDIAKSTRDRKRAYGALIASMDLQQNS
Eh_Ago2-1       IVSQITYQIQSKIGGIPWDVLTNNKFIKTMIIGIAVT--PYDDNKECISLVSSMKYNSFA
Eh_Ago2-2       VIQMITLQMQSKMGGIPWKTEIDSSFADGMTVGIDVI--SAGKDREIVAIVSSVD-KDFT
Eh_Ago2-3       ----LCLRTGIKLGCSRMKLDQNTSD--ICVIGITSR--EFFVNKTLITLCISKDENLN-
                    :  :   *:*          .      :*:          :    :  * .

Hs_Hiwi         RW-FSRCIFQDRGQELVDGLKVCLQAALRAWNSCNEYMPSRIIVYRDGVGDGQLKTLVNY
Dm_PIWI         TY-FSTVTECSAFDVLANTLWPMIAKALRQYQHEHRKLPSRIVFYRDGVSSGSLKQLFEF
Eh_Ago2-1       TRKKYSAIETKGSGNAGYNSQILIQKALDDWYKENLKYPSNIIIYRKANK-TSLLKIKEG
Eh_Ago2-2       VYKKSSVVEKKGLHTAGIHIGEFMKKALESYTEYNGSHPKKVIIYRGSANTGDLKNIQQG
Eh_Ago2-3       VFDTESYFVSNDNWHSGIGLEQILKPKL------EGRKLKRIFVYRCGISDNLMKAIRFG
                          .            :   *      .    ..:..** .     :  :

Hs_Hiwi         EVPQFLDCLKSIGR---GYNPRLTVIVVKKRVNTRFFAQSGGRLQNPLPGTVIDVEVTRP
Dm_PIWI         EVKDIIEKLKTEYARVQLSPPQLAYIVVTRSMNTRFFLNG----QNPPPGTIVDDVITLP
Eh_Ago2-1       ELNLLKQIINSK-----SISIKLLYFIVSCRNDIKVFSQNYQSYFNPQPGTIICDTIFEH
Eh_Ago2-2       ELVEVKKAITEY-----DQSIQFIYITVNNKHDMKFFSKDGNNFINPLPGTVITQGVTKT
Eh_Ago2-3       EVEQFLKICKQSC----GEEVKVCFITTD-NISSAIAIEENGELKSTKEGTFVISPLVNK
                *:  . .  .           :.  : .    .  .  :      .   **.:   :

Hs_Hiwi         EWYDFFIVSQAVRSGSVSPTHYNVIYDNSG-LKPDHIQRLTYKLCHIYYNWPGVIRVPAP
Dm_PIWI         ERYDFYLVSQQVRQGTVSPTSYNVLYSSMG-LSPEKMQKLTYKMCHLYYNWSGTTRVPAV
Eh_Ago2-1       DNFEFYLISHIPIKGVVRPVLYTSLYNSTE-FTQQQIYQITFDLCHLHYSVLSSIYTPAH
Eh_Ago2-2       DVFQFYLVSHVPRKGLVKPSMYFVLENTIENLSQLSLYNFTYELTHLYFNGANSVCFPTP
Eh_Ago2-3       NVKGFILNA---KPEICKTLKYVVLYDNTG-LTLREIIQFTFDNCHLSQGKMSTGKLPYC
                :   * : :            *  : ..   :.   : .:*:.  *:  .  .    *

Hs_Hiwi         CQYAHKLAFLVGQSIH
Dm_PIWI         CQYAKKLATLVGTNLH
Eh_Ago2-1       LFEANELSHVLNKCGY
Eh_Ago2-2       LYQANKFCKMLNDCGY
Eh_Ago2-3       ANEAKHFQKRLYS---
                   *:.:   :
```
