# Supplementary material for: Functional Characterization of Entamoeba histolytica Argonaute Proteins Reveals a Repetitive DR-Rich Motif Region That Controls Nuclear Localization
Source: mSphere. 2019 Oct 16;4(5):e00580-19. doi: 10.1128/mSphere.00580-19 (PMC6796981; doi:10.1128/mSphere.00580-19)
Supplement: TABLE S1 [file mSphere.00580-19-st001.pdf]

Suppl. Table I All oligonucleotides used in this study are listed

Primer name	Sequence
Ago2-1 Sma I Fwd	CCCGGGATGTTAAGTATTTATCC
Ago2-1 XhoI Rvs	CTCGAGTTAATAATAAGGATGTGAAC
Ago2-3 SmaI Fwd	CCCGGGATGAAACAAGTTGGAG
Ago2-3 XhoI Rvs	CTCGAGTTAATTCATTAGTTCTTC
Ago2-1 GA Fwd	GCTCTAAGAAAAAGCCCGGGATGTTAAGTATTTATCCAATTAAT
Ago2-1 GA Rvs	GAAGAGTTCAACTCGAGTTAATAATAAGGATGTGAACTAATA
Ago2-1 AA Fwd	ATATCATTTATAGATGCTGCTAAGTCAAAGTACGGA
Ago2-1 AA Rvs	TCCGTACTTTGACTTAGCAGCATCTATAAATGATAT
Ago2-3 GA Fwd	GCTCTAAGAAAAAGCCCGGGATGAAACAAGTTGGAGTATTAAGAGA
Ago2-3 GA Rvs	GAAGAGTTCAACTCGAGTTAATTCATTAGTTCTTCAAGTAT
Ago2-3 AA Fwd	CTGGGGAGATCAGGAAGTTTCTGTTTCACAAGCTGCTATGCAACGGT
Ago2-3 AA Rvs	TTGTGAAACAGAAACTTCCTGATCTCCCCAG
Ago2-2 GA Fwd	GCTCTAAGAAAAAGCCCGGGATGCAACCATCAATTCACGATTACTTCT
Ago2-2 GA Rvs	GAAGAGTTCAACTCGAGTTAGTGATGATGATCTCTATCATCTCTT
Ago2-2 AA Fwd	ACTCGTAAAGATGGAAGTTCTGTTTCTATTGCTGAAGCTGCTAAACAACGTT
Ago2-2 AA Rvs	AACGTTGTTTAGCAGCTTCAGCAATAGAAACAGAACTTCCATCTTTACGAGT
Ago2-2 del-NLS-DR Rvs	CTCGAGTCAACCTTCAACATAC
Ago2-2 del-DR Rvs	CTCGAGCTATCCTCCACCGAAGC
SmaI DHFR Fwd	AACCCGGGATGATCAGTCTGATTGCGGCGTT
Smal I DHFR Rvs	AACCCGGGCCGCTCCAGAATCTCAAAGCAATAG
Ago2-3 AvrII Rvs	AACCTAGGATTCATTAGTTCTTCAAGTATTTTAGA
Ago2-2 AvrII Fwd	GATTGTGGGTATGTTGAAGGTCCTAGGAAATCAAGTAGAGGAGGAAAT
Ago2-2 AvrII Rvs	ATTTCCTCCTCTACTTGATTTCCTAGGACCTTCAACATACCCACAATC
Ago2-2 D mut Fwd	ACAGTTGGTATTGCTGTTATTTCAG
Ago2-2 D mut Rvs	TGCCTGCTGAAATAACAGCAATACCAACTGT
Ago2-2 N mut Fwd	GTATCAAGCTGCTAAATTCTGTAAAATG
Ago2-2 N mut Rvs	CATTTTACAGAATTTAGCAGCTTGATAC
