# Supplementary material for: Functional Characterization of Entamoeba histolytica Argonaute Proteins Reveals a Repetitive DR-Rich Motif Region That Controls Nuclear Localization
Source: mSphere. 2019 Oct 16;4(5):e00580-19. doi: 10.1128/mSphere.00580-19 (PMC6796981; doi:10.1128/mSphere.00580-19)
Supplement: TABLE S2 [file mSphere.00580-19-st002.pdf]

Suppl. Table II Listed are Pfam domain analysis results for proteins with RRDDRD motif.

Pfam	Accession	Number of domains
RPEL	PF02755.15	147
PCI	PF01399.27	52
Helicase_C	PF00271.31	13
DEAD	PF00270.29	12
PAH	PF02671.21	12
Myb_DNA-binding	PF00249.31	8
FF	PF01846.19	8
MORN	PF02493.20	8
RRM_1	PF00076.22	7
Piwi	PF02171.17	7
PAZ	PF02170.22	5
Sin3_corepress	PF08295.12	4
Sin3a_C	PF16879.5	4
KH_1	PF00013.29	3
WW	PF00397.26	3
S1	PF00575.23	3
HA2	PF04408.23	3
CTD_bind	PF04818.13	3
COMPASS-Shg1	PF05205.12	3
SH3_2	PF07653.17	3
OB_NTP_bind	PF07717.16	3
Tho2	PF11262.8	3
Thoc2	PF11732.8	3
AAA_33	PF13671.6	3
THOC2_N	PF16134.5	3
SF1-HH	PF16275.5	3
